# Comparison of transverse and modified subtrochanteric femoral shortening osteotomy in total hip arthroplasty for developmental dysplasia of hip: a meta-analysis

**DOI:** 10.1186/1471-2474-15-331

**Published:** 2014-10-03

**Authors:** Changchuan Li, Chi Zhang, Maolin Zhang, Yue Ding

**Affiliations:** Department of Orthopaedic Surgery, Sun Yat-sen Memorial Hospital, No.107 on Yanjiangxi Road, Yuexiu District, 510120 Guangzhou, Guangdong China

**Keywords:** Subtrochanteric femoral shortening osteotomy, Total hip arthroplasty, Developmental dysplasia of the hip, Post-operative outcome, Meta-analysis

## Abstract

**Background:**

Subtrochanteric femoral shortening osteotomy is a crucial procedure to prevent nerve injury in total hip arthroplasty for severe developmental dysplasia of the hip. Transverse osteotomy was first applied, and other modified methods have also been reported. Each has its own advantages and limitations, but no definitive conclusions regarding differences in outcomes have been reached to date.

**Methods:**

We therefore performed a comprehensive meta-analysis to compare the outcomes of different approaches. 37 studies (795 hips) were included in the final analysis. Meta-analysis, subgroup analysis and meta-regression were performed.

**Results:**

Meta-analysis and subgroup analysis showed no significant difference between transverse and modified method. This is further confirmed by meta-regression. Method of osteotomy was found to be not associated with nonunion rate (*P* = 0.472), as well as other post-operative outcomes including nerve palsy (*P* = 0.240), dislocation (*P* = 0.735), revision (*P* = 0.653) and Harris hip score improvement (*P* = 0.562). In addition, western countries (*P* = 0.010) and duration of follow-up more than 5 years (*P* = 0.014) were associated with higher revision rate.

**Conclusions:**

Transverse osteotomy and modified osteotomy appear to be equivalent in terms of nonunion, safety and efficacy. Transverse osteotomy may be recommended, due to its simplicity and convenience in adjusting the anteversion angle. Well-designed and large-sample-size randomized controlled trials are expected to confirm and update the findings of this analysis.

**Electronic supplementary material:**

The online version of this article (doi:10.1186/1471-2474-15-331) contains supplementary material, which is available to authorized users.

## Background

Developmental dysplasia of the hip (DDH), formerly defined as congenital dislocation of the hip, is one of the most common neonatal deformities that may have significant influence on the life quality of patient [1]. Its incidence is estimated to be 3 to 5 per 1000 hips, which is clouded by the absence of definitive diagnostic criteria and the wide range of mild to severe anatomical variations that fall within the spectrum of DDH [2]. Although several options exist including proximal femoral and periacetabular osteotomies, total hip arthroplasty (THA) remains the standard treatment in end stage DDH, predominantly Crowe type IV in Crowe classification [3] or high dislocation DDH in Hartofilakidis classification [4], which leads to significant pain and loss of function [5].

Severely dysplastic hips present challenging surgical problems. The formation of a false acetabulum superior to the true acetabulum may lead to the need of leg lengthening, during the operation to get the center of rotation more anatomically [3, 6]. However, leg lengthening over 3–4 cm is associated with an increased risk of sciatic nerve injury [7]. Femoral shortening osteotomy has become a standard approach to avoid nerve injury [8]. Compared with great trochanter osteotomy, subtrochanteric femoral shortening has been more commonly used, because it has lower nonunion rate of osteotomy [9], and preserves the proximal femoral metaphysis, and thus allows for correction of rotation and the use of an uncemented femoral component [10]. On the other hand, it also provides correction of the excessive anteversion and lateral location of the abductor lever [10–12].

**Figure 1:**
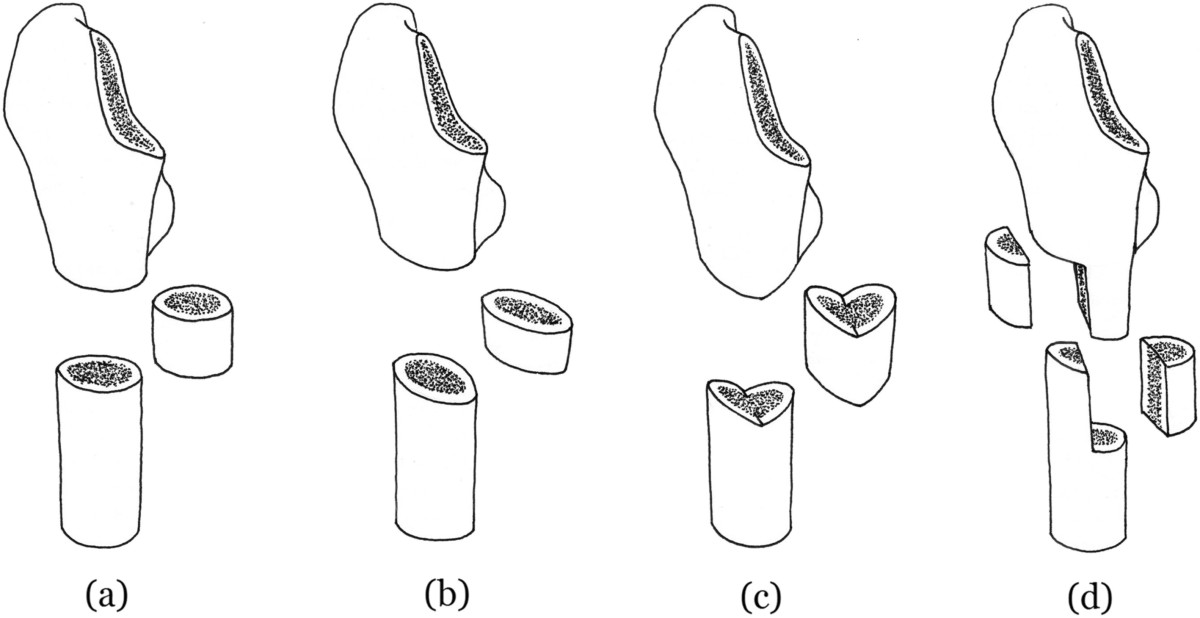
**Schematic illustration of different methods of subtrochanteric femoral shortening osteotomy. (a)** transverse **(b)** oblique **(c)** double-chevron **(d)** step-cut.

There are various techniques for subtrochanteric femoral shortening osteotomy, in attempt to decrease nonunion of osteotomy, which is one of the most commonly reported complications [6]. Transverse subtrochanteric femoral shortening osteotomy was first performed in THA for DDH patients. Subtrochanteric femur was transversely dissected to remove the excessive length, and the both ends of osteotomy were connected (Figure 1a). Due to the centrosymmetry of intersecting surface, adjustment of the two fragments of transverse osteotomy during the surgery is possible, when the initial alignment is not ideal [13]. This is important for the correction of femoral anteversion, the most common and one of the most important anatomic abnormalities caused by DDH [6, 10, 14], which is correlated with postoperative hip function [14].

However, the centrosymmetry of intersecting surface in turn increases the rotational instability, which in combination with a smaller surface contact might lead to nonunion of the osteotomy [9]. Step-cut [15], oblique [14], and chevron type [16] subtrochanteric femoral shortening osteotomies, which we categorize as “modified osteotomy”, were introduced in attempt to enhance the rotational stability of the osteotomy, and to reduce the risk of rotational instability and non-union [17]. Compared with transverse osteotomy, the osteotomy lines are step-cut, oblique and double-V-shaped, respectively (Figure 1b-1d). So they were expected to have lower nonunion rate of osteotomy. Many researchers have published their results and opinions on this issue, but few of them conducted a head-to-head comparison. Moreover, single clinical trials are often underpowered and lack generalizability [18]. Up to date, no definitive conclusions regarding differences in outcomes have been reached. We therefore systemically searched the literatures currently available, and performed a comprehensive meta-analysis to compare the outcomes of the two methods.

## Methods

### Data source

We searched PubMed, Embase and Cochrane Library up to August, 2014 for literatures which focused on subtrochanteric femoral shortening osteotomy for DDH and specified the method of osteotomy and the outcomes, using the following terms: ((((((congenital) OR developmental)) AND hip) AND (((dysplasia) OR dislocation) OR dislocations))) AND ((subtrochanteric) AND ((osteotomy) OR osteotomies)) (see Additional file 1). There was no restriction to regions or languages. The computer search was supplemented with manual searches of the reference lists of all retrieved literatures. When there were two or more reports describing the same population, the most recent or complete version was involved.

### Study eligibility and selection

The studies have to meet the following pre-determined inclusion criteria:

1. It investigated the subtrochanteric femoral shortening osteotomy in the surgical treatment of DDH, and short- and long-term outcomes of the surgery.
2. It provided data that allowed for quantitative analysis.
3. It had been published or accepted for publication.

Literatures that failed to meet the inclusion criteria were excluded. Besides, our exclusion criteria included:

1. Case report or review;
2. Not clinical studies (e.g. biomechanical);
3. Data of interest are not clear, and couldn’t be obtained by contacting the authors;
4. Duplicated data.

Two reviewers (C. Li and M. Zhang) independently evaluated the eligibility of involved studies. Discrepancies between evaluators were resolved by discussion or consultation with the corresponding author (Y. Ding).

### Data abstraction

Study characteristics were retrieved including author, year of publication, country, demographics of the population, method of subtrochanteric femoral shortening osteotomy, duration of follow-up, and union of the osteotomy sites. Data were extracted independently and in duplicate by both reviewers (C. Li and C. Zhang). Discrepancies between evaluators were resolved by discussion or consultation with the corresponding author (Y. Ding).

### Outcomes of interest

The following outcomes were used to compare transverse and modified subtrochanteric osteotomy:

### Primary outcomes

1. Nonunion (permanent failure of bone healing without treatment, usually identified at 8 months postoperatively). In consideration that delayed union might also require a revision surgery sometimes, like the case of nonunion, we also included delayed union that required a revision surgery into the category of nonunion.
2. Nerve palsy (transient or permanent, predominantly caused by stretching, could happen to sciatic nerve, femoral nerve, and occasionally to peroneal nerve).

### Secondary outcomes

Dislocation (early or recurrent), revision (due to all causes), leg-length discrepancy (average leg-length discrepancy, and discrepancy within ideal range, which was defined as 0-2 cm), Harris hip score (HHS) improvement (proportion of the difference between post- and pre-operative HHS in the post-operative HHS) and deep infection.

### Statistical analysis

This meta-analysis was performed according to the recommendations of Preferred Reporting Items for Systematic Reviews and Meta-Analyses [19]. Raw rates of outcome events were calculated in each study. As the inverse variance weight in fixed-effect meta-analysis is suboptimum when dealing with binary data with low probability, the variances of the raw rates were stabilized using Freeman-Tukey double-arcsine transformation [20]. In the meantime, the double-arcsine transformation could avoid the situation where the rate and standard error of a certain outcome is zero, which is not allowed for meta-analysis. All outcomes were reported with 95% CIs. Wilson’s method was used to calculate the 95% CI of the estimated rate to construct the forest plot, because the asymptotic method may produce confidence intervals that extend below zero, especially when the rates are estimated to be low.

We estimated heterogeneity between studies with Cochrane’s Q (reported as *χ*^*2*^ and *P* values), which is calculated as the weighted sum of squared differences between individual study effects and the pooled effect across studies, and the *I*^*2*^ statistic, which describes the percentage of variation between studies that is due to heterogeneity rather than chance [21]. *I*^*2*^ values of 25%, 50% and 75% are taken to indicate low, moderate and high degrees of heterogeneity, respectively. When the *I*^*2*^ statistic didn’t exceed 50%, we selected the fixed effects model, which could achieve higher statistical power than random effects model. Otherwise, random effects model is adopted. Sensitivity analysis was carried out to judge the weight of each study. After the meta-analysis of transformed data, we inversed the pooled estimate and its 95% CI back to proportions [22]. Up to date, there is no widely accepted scoring system for assessing the methodological quality of observational studies with no control.

Potential sources of heterogeneity were explored further by meta-regression analysis. The factors investigated in meta-regression included method of osteotomy (by comparing transverse and modified osteotomy), country (by comparing western and eastern countries), mean age (as a continuous variable, and then by dichotomizing the studies by the median of 49 years), proportion of female patients (as a continuous variable, and then by dichotomizing the studies by the median of 93%), duration of follow-up (as a continuous variable, and then by dichotomizing the studies by the median of 5 years), and year of publication (by dichotomizing the studies by the median of the year 2010). Categorical variables were taken into meta-regression using dummy variables [23].

All analyses were performed using STATA statistical software package (Version 13.0, StataCorp, 2013) using the commands cii (to calculate Wilson CIs), metan (for meta-analysis), metareg (for meta-regression) and metabias (to assess the publication bias). Generally, a *P* value <0.05 was considered to indicate statistical significance (α = 0.05).

## Results

### Characteristics of eligible studies

**Figure 2:**
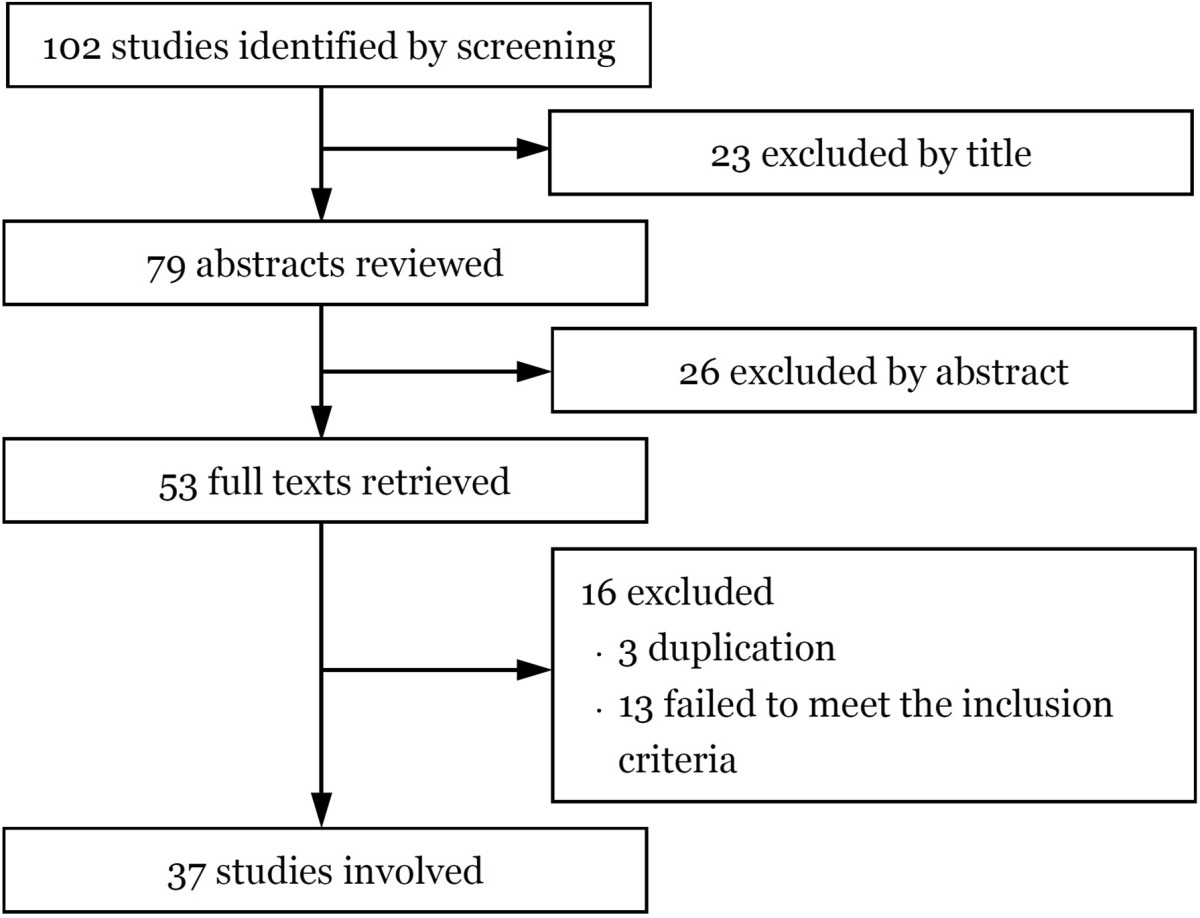
**Flowchart of the study selection process.**

37 studies (791 hips) were included in the final analysis (Figure 2). None of them was randomized controlled trial, case–control study or cohort study. The characteristics of the included studies are listed in Table 1. Agreement between the two reviewers was achieved.

**Table 1:** **Characteristics of included studies**

Study	Country	Mean age (range), year	Sex, Female %	Crowe classification	Total, hips/patients	Method of osteotomy	Mean follow-up (range), years	Nonunion, hips	Nerve injury, hips	Revision	Dislocation	Deep infection	Harris hip score		Limb-length discrepancy		Acceptable discrepancy, patients
													Pre-operative	Post-operative	Pre-operative	Post-operative	
Becker, 1995	USA	61 (48–72)	N/A	IV	7/4	Double-chevron	2.7 (0.3-6)	0	0	1	0	N/A	N/A	N/A	N/A	N/A	N/A
Reikeraas, 1996	Norway	54 (17–67)	93.8	IV	25/19	Transverse	(3–7)	0	1	N/A	N/A	N/A	43	93	N/A	N/A	N/A
Yasgur, 1997	USA	42 (22–77)	77.8	IV	9/8	Transverse	3.6 (2–7)	1	0	1	1	N/A	N/A	N/A	N/A	1.5	N/A
Chareancholvanich, 1999	USA	51 (21–74)	N/A	N/A	15/11	Double-chevron	5.5 (2–8.5)	0	0	N/A	0	N/A	N/A	N/A	3.9	1.4	N/A
Zadeh, 1999	UK	49 (34–61)	71.4	I, II & IV	7/7	Transverse	2.6 (1.3-5.0)	1^*^	N/A	N/A	N/A	N/A	44	91	N/A	N/A	N/A
Bruce, 2000	Australia	53 (26–77)	N/A	III & IV	6/5	Transverse	4.7 (0.5-7.2)	0	0	N/A	N/A	0	31	81	N/A	N/A	N/A
Ozturkmen, 2002	Turkey	N/A	N/A	IV	7/7	Step-cut	N/A	1	N/A	N/A	N/A	N/A	N/A	N/A	N/A	N/A	N/A
Sener, 2002	Turkey	43 (26–64)	95.7	III & IV	28/23	Step-cut	4.0 (0.6-7.7)	2	2	0	0	0	36.9	95.3	N/A	N/A	19
Decking, 2003	Germany	47 (19–58)	70.0	II.III & IV	12/10	Step-cut	5.1 (1.6-10)	0	1	1	0	0	36	82	5.4	1.3	N/A
Masonis, 2003	Canada	48.2 (21–70)	84.2	III & IV	21/19	Transverse	5.8 (2.0-11.2)	2	0	3	3	0	32.5	73.6	N/A	N/A	N/A
Erdemli, 2005	Turkey	44 (28–61)	100.0	IV	25/22	Step-cut in 3 hips	5 (2–10)	0	0	N/A	N/A	0	N/A	N/A	N/A	N/A	N/A
						Transverse in 22 hips		1	0	N/A	N/A	0	N/A	N/A	N/A	N/A	N/A
Bernasek, 2007	USA	43 (17–67)	91.3	I.II.III & IV	23/20	Transverse	8 (5–14)	0	0	1	4	0	42	82	N/A	N/A	N/A
Gotze, 2007	Germany	41.7 (29–64)	N/A	III & IV	7/7	Transverse	1.5	0	N/A	N/A	N/A	N/A	43	77	N/A	N/A	N/A
Makita, 2007	Japan	59.6 (42–76)	100.0	IV	11/11	Step-cut	5.4 (2.5-14.1)	0	2	1	1	0	N/A	N/A	4.7	1.2	7
Park, 2007	Korea	44.8 (20–66)	N/A	III & IV	24/23	Transverse	4.7 (2.0-7.6)	3	0	1	1	N/A	35.6	81.7	N/A	N/A	21
Krych, 2009	USA	N/A	N/A	IV	28/24	Transverse	4.8	2	0	N/A	4	N/A	43	89	N/A	N/A	N/A
Nagoya, 2009	Japan	55 (44–69)	94.4	IV	20/18	Transverse	8.1 (4–11.5)	0	0	2	0	0	N/A	N/A	N/A	1.2	N/A
Howie, 2010	UK	47.3 (26–75)	N/A	III & IV	35/28	Transverse	5.6 (2–14)	1	2	7	3	1	N/A	N/A	N/A	N/A	N/A
Reikeraas, 2010	Norway	48 (16–79)	70.8	III & IV	65/46	Transverse	13 (8–18)	2	2	11	1	N/A	N/A	87	N/A	1.0	N/A
Togrul, 2010	Turkey	42.3 (33–52)	85.7	III & IV	21/14	Transverse	3.4 (2.0-5.3)	0	0	N/A	2	0	N/A	N/A	N/A	0.3	N/A
Akiyama, 2011	Japan	58.9 (42–77)	90.9	III & IV	15/11	Transverse	6.3 (2.8-10.4)	3	0	3	2	N/A	N/A	N/A	2.9	0.3	11
Charity, 2011	UK	51 (33–75)	100.0	IV	18/15	Transverse	9.5 (4.3-14)	1	1	4	0	N/A	N/A	N/A	N/A	N/A	N/A
Dallari, 2011	Italy	52 (34–66)	76.9	IV	33/26	Step-cut in 14 hips	7.3 (2.2-15.3)	0	N/A	N/A	N/A	N/A	N/A	N/A	7.0	3.0	N/A
						Oblique in 19 hips		1	N/A	N/A	N/A	N/A	N/A	N/A			N/A
Kawai, 2011	Japan	64.8 (57–73)	100	IV	19/12	Transverse	3.2 (0.5-8)	0	0	N/A	0	0	N/A	N/A	N/A	N/A	N/A
Kilicarslan, 2011	Turkey	46 (20–72)	N/A	III & IV	45/31	Transverse	7.2 (2.0-10.1)	5	0	N/A	2	N/A	N/A	N/A	2.9	1.4	N/A
Starker, 2011	Germany	44.6	83.3	IV	25/20	Step-cut	N/A	0	N/A	N/A	N/A	N/A	N/A	90	N/A	0.8	20
Takao, 2011	Japan	60	92.0	IV	33/25	Step-cut	8 (5–11)	0	0	1	2	N/A	N/A	N/A	5.1	2.8	N/A
Zhong, 2011	China	45.2 (36–56)	100.0	IV	36/28	Transverse	4.4 (2.3-7.8)	0	0	N/A	0	0	39	87	5.7	0.6	N/A
Baz, 2012	Turkey	41.6 (24–56)	86.7	IV	21/15	Transverse	4.9 (3–8)	0	1	2	2	0	36.2	90.8	N/A	N/A	N/A
Hasegawa, 2012	Japan	58.5 (48–72)	100.0	IV	15/N/A	Step-cut	10.2 (5–20)	0	1	4	3	0	56	85	3.8	1.4	N/A
Semenowicz, 2012	Poland	53.4	100.0	IV	10/10	Step-cut	2.3	0	0	N/A	N/A	N/A	43.7	86	N/A	N/A	N/A
Kilicoglu, 2013	Turkey	43 (27–60)	95.0	IV	20/16	Oblique	6.8 (3.7-10.3)	1^*^	0	2	3	N/A	50	83	N/A	1.0	N/A
Li WB, 2013	China	N/A	100	IV	5/5	Transverse	N/A	0	0	0	0	N/A	N/A	N/A	N/A	N/A	N/A
Li YW, 2013	China	54 (41–75)	83.3	IV	22/18	Double-chevron	8 (3–12)	0	0	0	0	0	30	91.9	2.5	1	18
Oe, 2013	Japa	64.9 (35–80)	96.2	IV	34/26	Transverse	5.2 (3–10)	0	0	0	3	0	N/A	N/A	4.7	1.2	26
Sun, 2013	China	47 (38–65)	63.3	IV	32/27	Transverse	4 (0.6-7)	0	1	0	0	0	41.7	89.1	N/A	N/A	N/A
Oinuma, 2014	Japan	61.5 (46–73)	100	IV	12/9	Transverse	3.7 (1.5-6.3)	0	0	0	1	0	N/A	N/A	N/A	N/A	N/A
Total	/	50.0	88.1	/	791/N/A	/	/	27	14	45	38	1	/	/	/	/	/

N/A: not available.

*Delayed union that needs a second procedure.

Of the 37 studies enrolled in the meta-analysis, 10 were conducted in Europe [11, 12, 14, 15, 24–29], 6 in the North America [7, 16, 30–33], 1 in Australia [34], and the other 20 in Asia [9, 13, 17, 35–51]. Female took the majority of the patients, and 9 studies were based on female patients exclusively.

Most researchers have used only one method of subtrochanteric osteotomy in their own studies, however, with two exceptions. Erdemli et al. [35] applied step-cut osteotomy in 3 hips, and transverse osteotomy in 22 hips. The other researcher, Dallari [14], performed step-cut osteotomy in 14 hips, and oblique osteotomy in 19 hips. These two studies were split in meta-analysis and meta-regression. The transverse group included 24 studies (550 hips), and the modified group included 15 studies (241 hips).

### Primary outcomes

#### Nonunion

**Figure 3:**
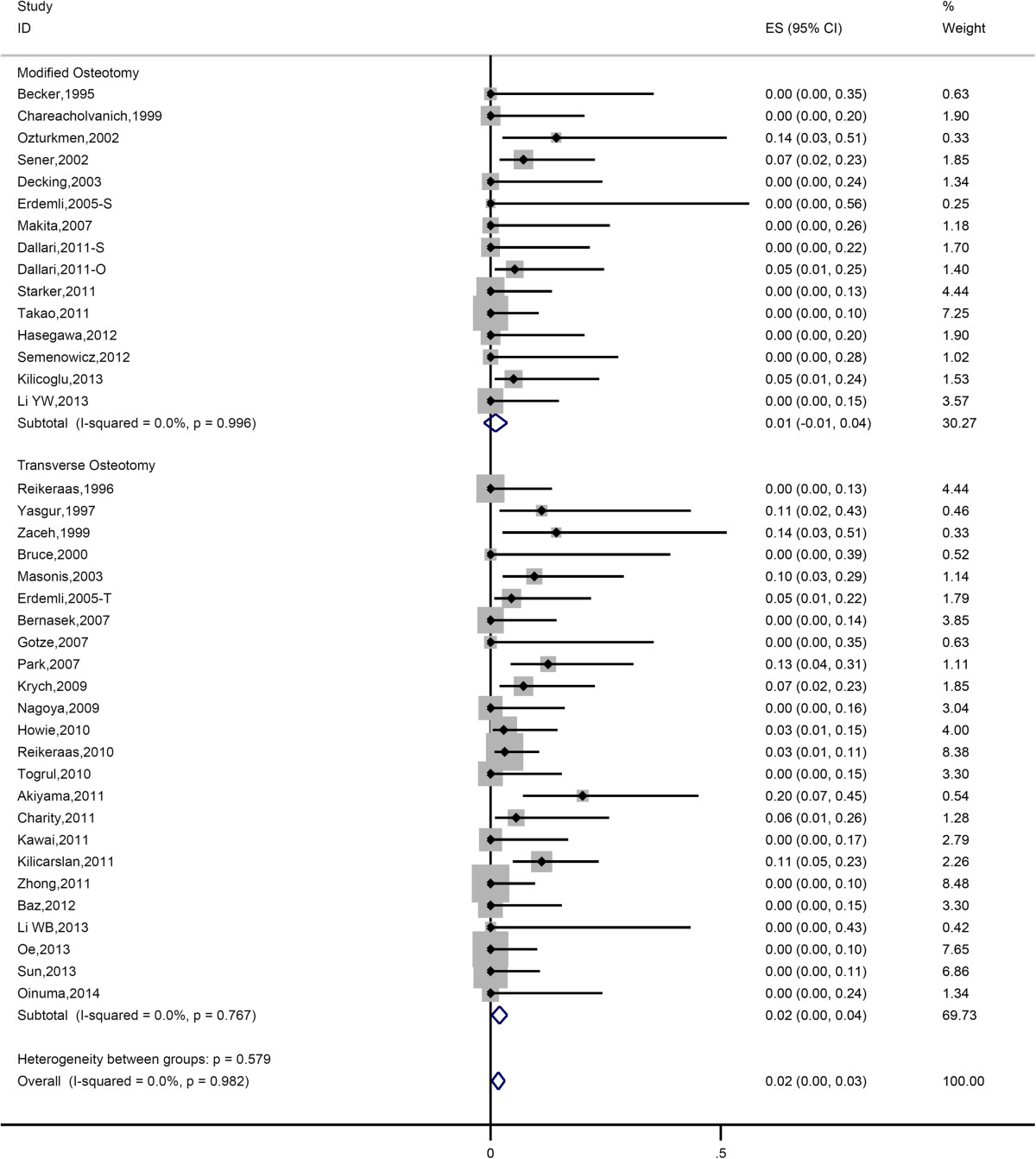
**Forest plot of all eligible studies for nonunion rate.**

Pooled estimate of nonunion rate was 3.79% (95% CI 2.60%-5.20%). Heterogeneity among the studies was low (*I*^*2*^ = 14.5%). Data of nonunion were further analyzed in two subgroups (modified osteotomy and transverse osteotomy). Figure 3 showed the forest plot of subgroup analysis. There was no significant difference in nonunion rate between modified group and transverse group (Table 2).

**Table 2:** **Outcomes of meta-analysis and subgroup analysis**

Outcome of interest	No. of studies	No. of hips	No. of events	Pooled estimate	95% CI	Study heterogeneity		
						χ^2^	I^2^	*P* value
Nonunion	39	791	27	3.79%	(2.60%, 5.20%)	39.25	3.2%	0.414
Modified subgroup	15	241	5	3.03%	(1.29%, 5.51%)	8.29	0.0%	0.874
Transverse subgroup	24	550	22	4.14%	(2.67%, 5.20%)	30.34	24.2%	0.140
Nerve palsy	33	712	14	2.63%	(1.60%, 3.87%)	21.9	0.0%	0.910
Dislocation	27	641	38	5.88%	(4.22%, 7.80%)	42.35	36.2%	0.030
Modified subgroup	9	163	9	5.47%	(2.57%, 9.36%)	12.99	38.4%	0.112
Transverse subgroup	18	478	29	6.03%	(4.14%, 8.29%)	29.28	38.5%	0.045
Revision	22	482	45	8.90%	(6.56%, 11.50%)	45.57	53.9%	0.001
Modified subgroup	8	148	10	6.66%	(3.31%, 11.12%)	14.04	50.1%	0.050
Transverse subgroup	14	334	35	9.96%	(7.06%, 13.35%)	29.99	56.7%	0.005
Limb-length discrepancy	7	132^*^	10^*^	6.31%	(2.90%, 11.00%)	18.37	67.3%	0.005
Modified subgroup	4	72	8	9.02%	(3.64%, 16.40%)	13.86	78.4%	0.003
Transverse subgroup	3	60	2	3.72%	(0.50%, 9.72%)	2.83	29.3%	0.243
HHS improvement	17	337	/	26.79%	(26.35%, 27.32%)	654.87	97.6%	<0.001
Modified subgroup	6	107	/	27.41%	(26.52%, 28.31%)	569.55	99.1%	<0.001
Transverse subgroup	11	230	/	26.61%	(25.99%, 27.14%)	82.56	87.9%	<0.001
Deep infection	19	393	1	1.34%	(0.46%, 2.67%)	2.24	0.0%	1.000

*Number of patients; CI: confidential interval.

#### Nerve palsy

**Figure 4:**
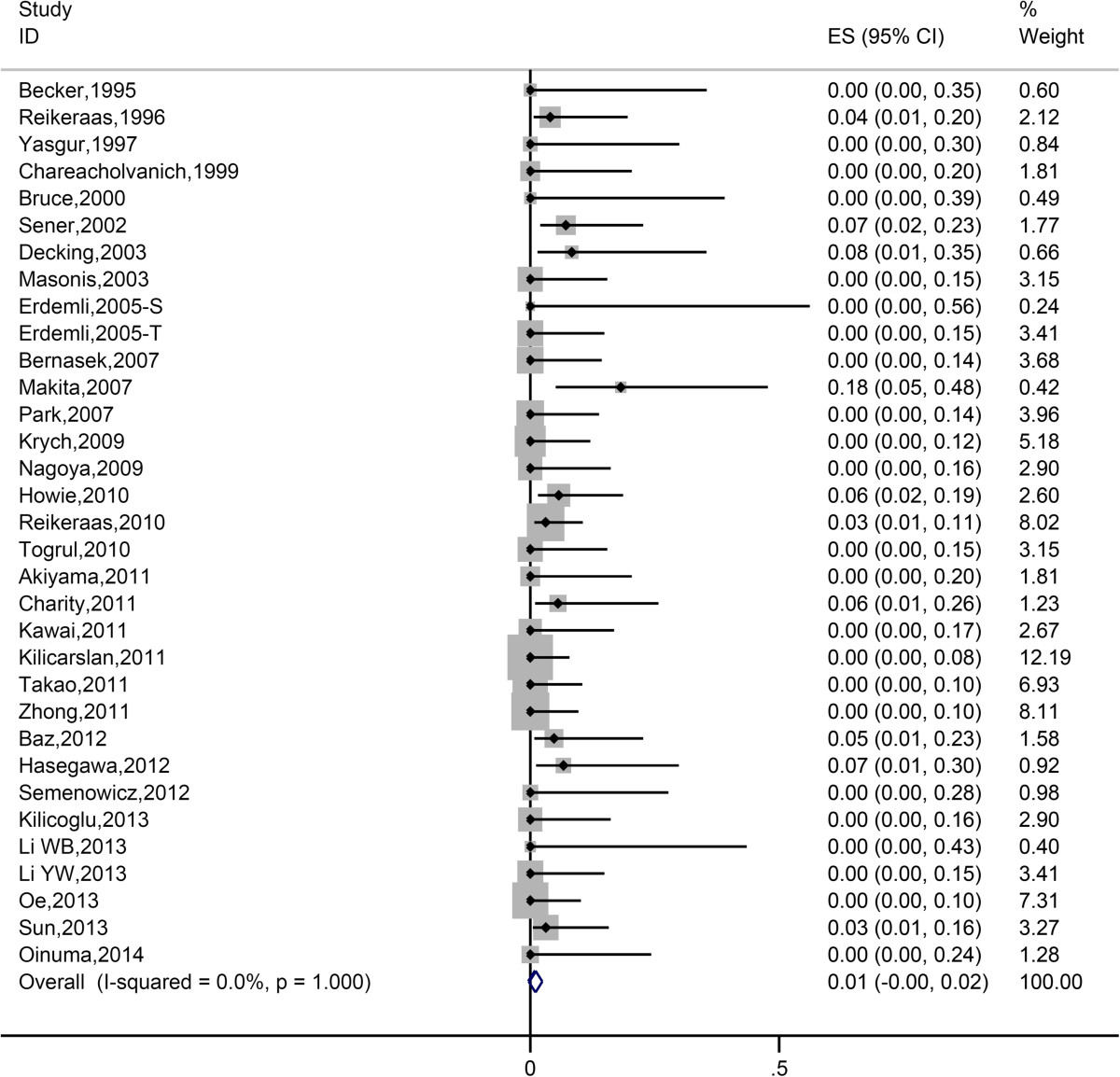
**Forest plot of all eligible studies for nerve palsy rate.**

Pooled estimate of nerve palsy rate was 2.63% (95% CI 1.60%-3.87%). Forest plot of meta-analysis was shown in Figure 4. According to Table 2, there was so little heterogeneity among the studies (*I*^*2*^<0.1%), that subgroup analysis was not necessary.

### Secondary outcomes

#### Dislocation

**Figure 5:**
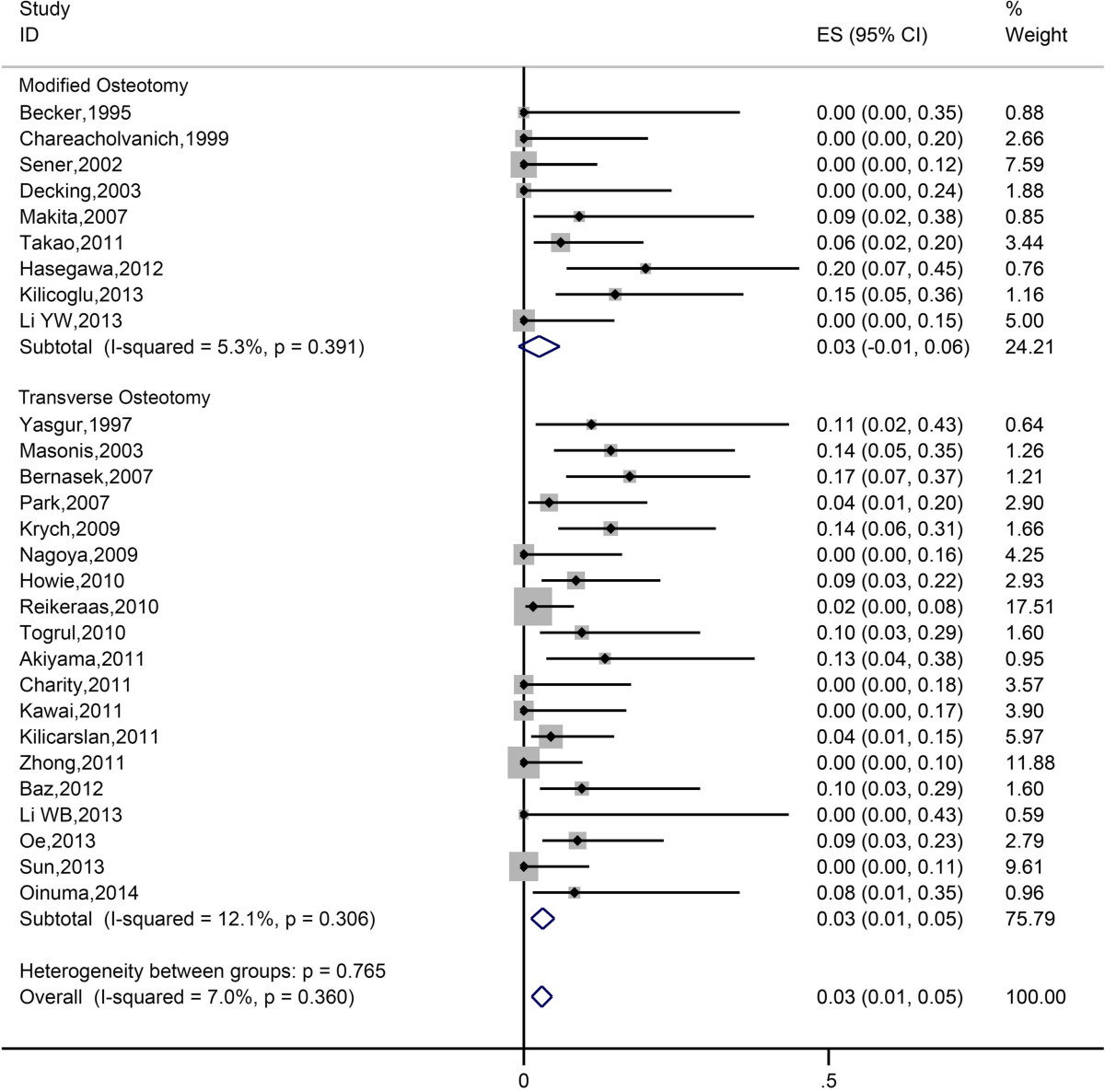
**Forest plot of all eligible studies for dislocation rate.**

Pooled estimate of dislocation rate was 5.88% (95% CI 4.22%-7.80%) Heterogeneity among the studies was moderate (*I*^*2*^ = 36.2%). Data of dislocation were further analyzed in subgroup analysis, and forest plot was constructed (Figure 5). No significant difference in dislocation rate was revealed between modified group and transverse group, and subgroup heterogeneity were not significantly less (*I*^*2*^ = 38.4% for modified subgroup and *I*^*2*^ = 38.5% for transverse subgroup) (Table 2).

#### Revision

**Figure 6:**
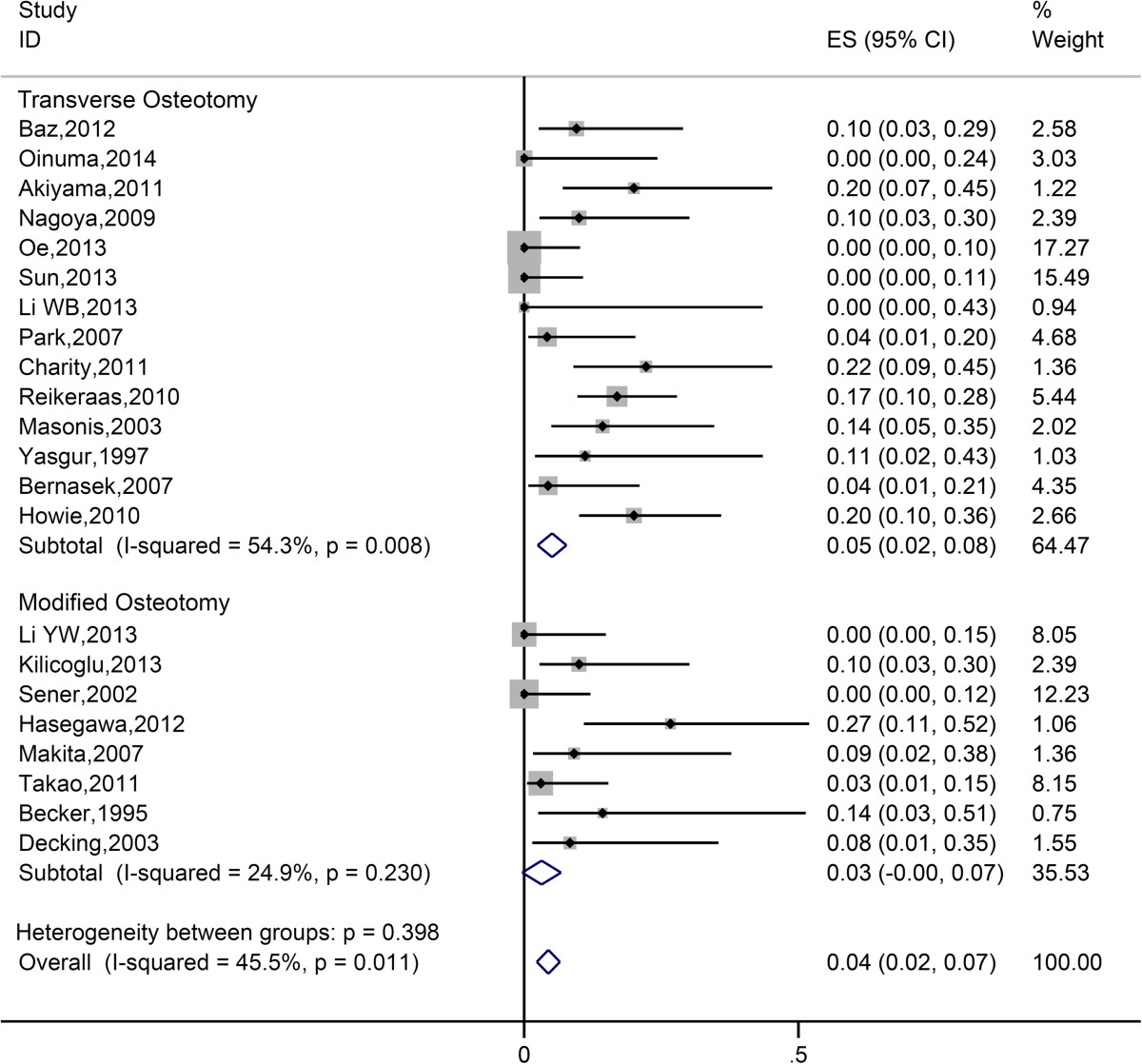
**Forest plot of all eligible studies for revision rate.**

Pooled estimate of revision rate due to all causes was 8.90% (95% CI 6.56%-11.50%). Heterogeneity among the studies was moderate (*I*^*2*^ = 43.3%). Data of revision were further analyzed in subgroups, and forest plot was constructed (Figure 6). As Table 2 indicated, no significant difference in revision rate was shown between modified group and transverse group.

#### Leg-length discrepancy

Only five studies provided related data on the number of patients who achieved ideal rage of leg-length discrepancy. Occurrence of discrepancy out of the ideal range was analyzed. Pooled estimate of occurrence was 6.31% (95% CI 2.90%-11.00%). Obvious heterogeneity existed among the studies (*I*^*2*^ = 67.2%). Subgroup analysis was carried out, revealing no significant difference between modified group and transverse group (Table 2).

#### HHS improvement

Average HHS in each of the eligible studies have elevated after the surgery. Heterogeneity among the studies was high (*I*^*2*^ = 96.5%). However, subgroup analysis revealed no significant difference between modified group and transverse group (Table 2).

#### Deep infection

Pooled estimate of deep infection rate was 1.34% (95% CI 0.46%-2.67%). There was so little heterogeneity among the studies (*I*^*2*^<0.1%), that subgroup analysis was not necessary (Table 2).

### Sensitivity analysis

Sensitivity analysis was carried out by excluding each study in turn to ensure that no single study would be solely responsible for the heterogeneity of any result. The results were almost the same as those when all studies were involved.

### Meta-regression

Univariate meta-regression analysis was carried out for outcomes of interest to explore potential influencing factors. Method of osteotomy was found to be not associated with nonunion rate (*P* = 0.472), as well as other post-operative outcomes including nerve palsy (*P* = 0.240), dislocation (*P* = 0.735), revision (*P* = 0.653) and Harris hip score improvement (*P* = 0.562) (Table 3).

**Table 3:** **Outcomes of meta-regression analysis**

	Nonunion		Nerve palsy		Revision		Dislocation		HHS improvement	
	*P*	Co.	*P*	Co.	*P*	Co.	*P*	Co.	*P*	Co.
Method of osteotomy (modified vs. transverse)	0.472	−0.030	0.240	0.051	0.653	−0.033	0.735	−0.020	0.562	−0.025
Country (western vs. eastern)	0.731	0.013	0.272	0.042	0.010	0.169	0.592	0.029	0.819	−0.010
Mean Age	0.141	−0.004	0.734	0.000	0.760	−0.001	0.919	0.000	0.736	−0.001
Mean Age (<49y vs. ≥ 49y)	0.302	0.042	0.958	−0.002	0.980	0.002	0.948	−0.004	0.940	0.004
Proportion of female	0.845	0.000	0.587	0.000	0.721	0.001	0.367	0.003	0.290	−0.002
Proportion of female (<93% vs. ≥ 93%)	0.645	0.019	0.833	−0.009	0.793	0.021	0.723	0.026	0.143	0.073
Follow up	0.906	0.000	0.700	0.003	0.074	0.021	0.937	0.000	0.408	−0.008
Follow up (≤5y vs.>5y)	0.937	0.003	0.871	−0.006	0.014	−0.163	0.391	−0.046	0.354	0.042
Year of publication (2010 or before vs. after 2010)	0.187	0.051	0.244	0.044	0.300	0.071	0.716	0.019	0.504	0.028

HHS: Harris hip score; Co.: meta-regression coefficient.

Results of the meta-regression on revision rate indicated that western countries and longer follow-up (>5y) are associated with higher revision rate (Table 3).

Meta-regression analysis was not performed for leg-length discrepancy, due to limited quantity of eligible studies. Similarly, there was only one case of deep infection among all the involved studies, and thus meta-regression was not performed for deep infection.

### Publication bias assessment

**Figure 7:**
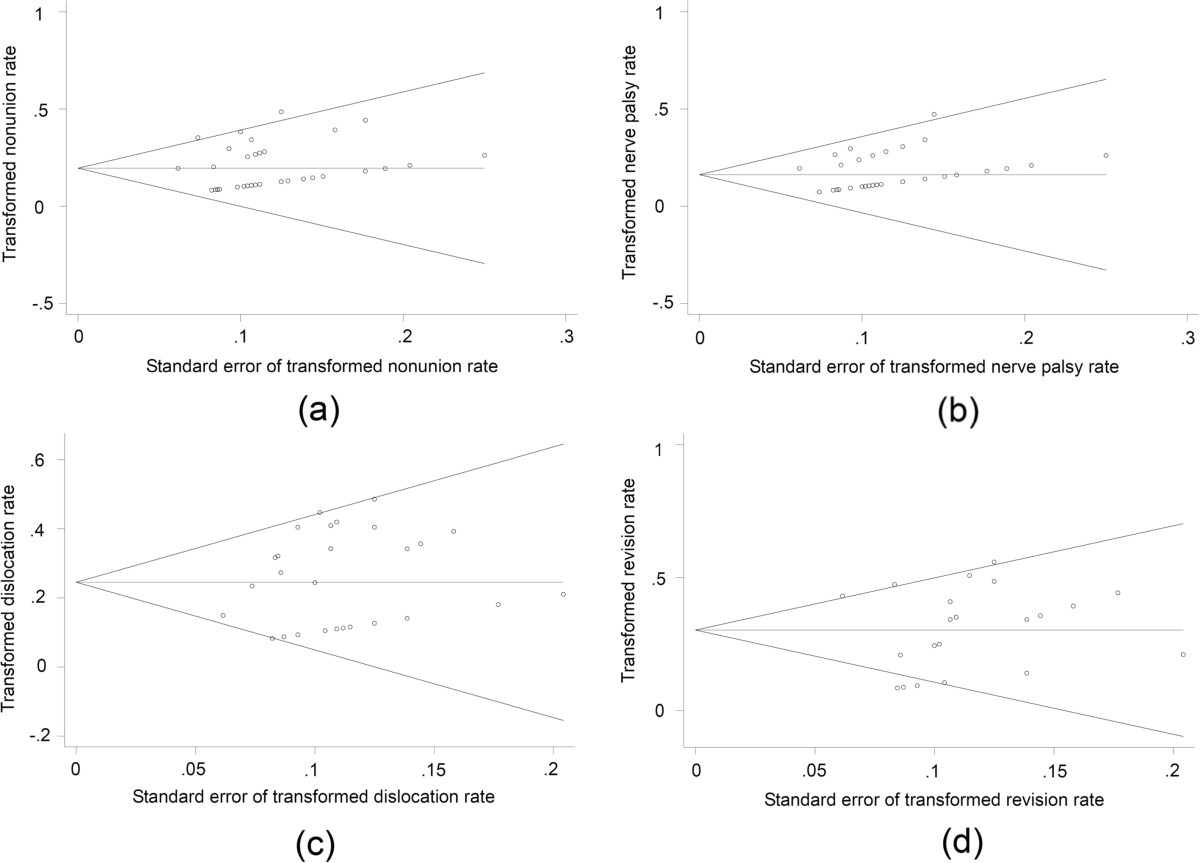
**Begg’s funnel plot of all eligible studies for (a) nonunion rate (b) nerve palsy (c) dislocation rate (d) revision rate.**

Begg’s funnel plots and Egger’s regression asymmetry tests were performed to investigate whether publication bias existed (Figure 7). All outcomes were distributed symmetrically in funnel plots, indicating no obvious publication bias. Egger’s regression asymmetry tests for nonunion, nerve palsy, revision, dislocation, leg-length discrepancy and HHS improvement showed no evidence of publication bias (*P* value was 0.380, 0.186, 0.714, 0.165, 0.524 and 0.393, respectively). *P* value of Egger’s test for deep infection was 0.044, indicating a potential publication bias.

## Discussion

This meta-analysis included 15 studies concerning modified osteotomy and 24 studies concerning transverse osteotomy. The results showed that method of osteotomy was not associated with nonunion rate, as well as other post-operative outcomes including nerve palsy, dislocation, revision, leg-length discrepancy, HHS improvement and deep infection. In addition, western countries and longer follow-up (>5.1y) were associated with higher revision rate.

According to our analysis, transverse osteotomy and modified osteotomy didn’t show significant difference in nonunion rate (Figure 3). The analysis also showed that transverse and modified osteotomy didn’t show significant difference in terms of occurrence of nerve palsy, indicating that they have equal efficacy in preventing post-operative complication concerning nerve stretching (Figure 4). This could be attributed to the fact that the occurrence of nerve palsy is predominantly associated with the extent of leg lengthening.

In addition, occurrence of other post-operative complication including dislocation and deep infection is similar between transverse osteotomy and modified osteotomy. Recognized risk factors of dislocation after total hip arthroplasty include age, sex, head diameter of femoral prosthesis, surgical approach and experience of surgeon [52]. It wasn’t unexpected that method of osteotomy had no influence on post-operative dislocation. As for the relationship between method of osteotomy and infection rate, there had been different opinions. Modified osteotomies seem to take more time than transverse osteotomies due to their complexity [45, 46, 49], and there were several studies showing that prolonged operation time would increase the risk of deep infection after total hip arthroplasty [53–55]. The deduction may seem reasonable that modified osteotomy would have higher infection rate than transverse osteotomy. However, our meta-analysis revealed that modified osteotomy and transverse osteotomy shared similar infection rate. This is probably because that the difference of operation time for modified and transverse osteotomy was not so significant to bring a difference in infection rate. On the other hand, some studies suggested that operation time might have no significant impact on deep infection rate at all [56, 57]. As for revision rate, there’s also no significant difference. In term of clinical improvement, modified osteotomy didn’t seem to show better performance than transverse osteotomy in post-operative leg-length discrepancy and HHS.

There was continuous suspicion that transverse osteotomy may not yield so good bone union as modified osteotomy, because of its lesser contact area for bone union, and potential rotational instability [9, 58]. However, our analysis showed that transverse osteotomy could yield similar bone union as modified osteotomy. In the meantime, transverse osteotomy equally improved hip function, and is equally safe in avoiding complications as modified osteotomy. And modified osteotomy is reported to be under the risk of arm fracture of osteotomy as a postoperative complication [14], which is impossible after transverse osteotomy. We concluded that transverse osteotomy share similar nonunion rate with modified osteotomy, and is equally safe and effective as modified osteotomy.

As a matter of fact, derotation for femoral anteversion is usually necessary, which in turn requires prompt preoperative preparation and extreme accuracy of osteotomy to correct the femoral anteversion [13, 17]. For modified osteotomy, once dissection is performed, there would be no chance for further adjustment of the anteversion angle of the femoral neck. Modified osteotomy also takes longer time than transverse osteotomy, due to the complexity of the surgical procedure, and the technically difficult procedure needs a lot of time and exercise for surgeons to master. Transverse osteotomy may be recommended, due to its simplicity and convenience in adjusting the anteversion angle.

Researchers have been trying to enhance the rotational stability of osteotomy, in order to improve bone union. Meanwhile, additional remarks should be made that rotational stability is influenced by not only the method of osteotomy, but also the design of implant used. Usually, DDH patient who receives THA is not so old to be free of risk of revision, and bone cement impedes later revision. On the other hand, possible leakage of bone cement would disturb the bone union. Taking these into consideration, researchers tended to perform cementless THA for DDH patients who needed subtrochanteric femoral shortening osteotomy at the same time. However, cross section and coating of femoral prostheses vary due to different designs. More importantly, press-fit femoral stem used in cementless THA mainly rely on proximal fixation, which might be insufficient to provide favorable stability for subtrochanteric osteotomy, for subtrochanteric osteotomy itself requires stable fixation of both proximal and distal femur, on metaphysis and diaphysis, respectively [43]. In recent years, some researchers applied modular femoral stem (e.g. S-ROM, DePuy) to these patients [33, 34, 43, 44, 46, 48], and there were few reports on nonunion among them. Due to its distinctive design, modular stem guarantees both proximal and distal fixation, and thus has potential advantages in providing rotational stability for subtrochanteric osteotomy. Nevertheless, more studies are expected before consensus is achieved whether modular stem facilitates better union of subtrochanteric osteotomy.

Further analysis indicated that western countries and longer follow-up (>5y) are associated with higher revision rate. Longer follow-up was to be expected on the basis of common sense and reasonable deduction. Western countries turned out to be associated with higher revision rate, probably due to its correlation with longer follow-up. Mean follow-up was 8.6 years in the studies of western countries, while 6.0 years in those of eastern countries.

This meta-analysis has the following limitations that must be taken into account. First, all the studies included were not randomized controlled trials, and thus of lower level of evidence. The studies were conducted with varying protocols and different levels of surgical expertise. Second, we compare transverse osteotomy and modified osteotomy, which is a category including step-cut, oblique and double-chevron osteotomy. There were much less studies on each of the modified osteotomy than on transverse osteotomy, which would result in significant loss of statistical efficacy if we compare each to transverse osteotomy separately. On the basis that the step-cut, oblique and double-chevron osteotomy shared some certain common ground, we categorized them into modified osteotomy in statistical analysis, as a strategy against potential loss of statistical efficacy. Third, the computer-based literature was systematic, and supplemented with manual searches. However, we may not be able to identify all the relevant studies despite our precise selection. In addition, the quantity of eligible studies was limited, and meta-regression analysis was not carried out for limb-length discrepancy due to lack of data.

To the best of our knowledge, this is the first meta-analysis comparing transverse and modified subtrochanteric femoral shortening osteotomy in THA for DDH patients. Second, it was conducted at an appropriate time, when enough data have accumulated for inspection by meta-analytical method. Third, this study was based on systematic up-to-date searching and filtering of literature, strictly according to the predetermined inclusion and exclusion criteria. Moreover, non-English language studies were included to minimize publication bias [36, 44, 46, 48, 49]. In addition, the variances of the raw rates were stabilized using Freeman-Tukey double-arcsine transformation, for the data were binary with low probability. Last but not least, it brings out a universal conclusion by including studies of various continents and countries. This meta-analysis therefore provides the most up-to-date information in this area.

## Conclusions

This meta-analysis indicated that transverse osteotomy shared similar nonunion rate with modified osteotomy, and was equally safe and effective as modified osteotomy. Transverse osteotomy may be recommended, due to its simplicity and convenience in adjusting the anteversion angle. Despite our rigorous methodology, the inherent limitations of the included studies are barriers for us to reach definitive conclusions. Well-designed and large-sample-size randomized controlled trials are expected to confirm and update the findings of this analysis.

## Electronic supplementary material

Additional file 1:A detailed description of the literature screening and filtering.(DOC 62 KB)

Below are the links to the authors’ original submitted files for images.

Authors’ original file for figure 1

Authors’ original file for figure 2

Authors’ original file for figure 3

Authors’ original file for figure 4

Authors’ original file for figure 5

Authors’ original file for figure 6

Authors’ original file for figure 7

Authors’ original file for figure 8

Authors’ original file for figure 9
