# Supplementary material for: Comparison of transverse and modified subtrochanteric femoral shortening osteotomy in total hip arthroplasty for developmental dysplasia of hip: a meta-analysis
Source: BMC Musculoskelet Disord. 2014 Oct 3;15:331. doi: 10.1186/1471-2474-15-331 (PMC4201680; doi:10.1186/1471-2474-15-331)
Supplement: Supplementary file 1 — Additional file 1:A detailed description of the literature screening and filtering.(DOC 62 KB) [file 12891_2014_2280_MOESM1_ESM.doc]

**Additional File**

**A detailed description of the literature screening and filtering**

We searched PubMed, Embase and Cochrane Library up to August, 2014 for literatures which focused on subtrochanteric femoral shortening osteotomy for DDH and specified the method of osteotomy and the outcomes, using the following terms: ((((((congenital) OR developmental)) AND hip) AND (((dysplasia) OR dislocation) OR dislocations))) AND ((subtrochanteric) AND ((osteotomy) OR osteotomies)). The computer search was supplemented with manual searches of the reference lists of all retrieved literatures. 102 studies were screened for eligibility to be involved in the meta-analysis. Detailed screening processes are as below:

**Excluded by title (23 studies)**

12, 35, 39, 40, 45, 50, 57, 64, 76, 78, 80-82, 84-93

**Excluded by abstract (26 studies)**

1, 2, 4, 8, 10, 13, 43, 48, 51, 52, 55, 59, 62, 65, 66, 67, 69, 70-75, 77, 79, 83

**Excluded by full-text (13 studies)**

3, 14, 15, 19, 20, 22, 23, 29, 34, 41, 42, 49, 56

**Duplicated (3 studies)**

30 (duplicate data of 36), 32 (duplicate data of 97), 98 (duplicate data of 47)

**Involved (37 studies)**

5, 6, 7, 9, 11, 16-18, 21, 24-28, 31, 33, 36- 38, 44, 46, 47, 53, 54, 58, 60, 61, 63, 68, 94-97, 99-102

**Exclusion criteria included**

1. Fail to meet inclusion criteria;
2. Case report or review;
3. Not clinical studies (e.g. biomechanical);
4. Data of interest are not clear, and couldn’t be obtained by contacting the authors;
5. Duplicated data.

Note: the numbers refer to the corresponding literatures in the list of references, which starts from the next page.

1. Surgical results of developmental dysplasia of the hip in older children based on using three-dimensional computed tomography. Zhao X, Yan YB, Cao PC, Ma YS, Wu ZX, Zhang Y, Zang Y, Jie Q, Lei W. J Surg Res. 2014 Jun 15;189(2):268-73. doi: 10.1016/j.jss.2014.03.003. Epub 2014 Mar 11. PMID: 24703507.
2. [Autograft of femoral head for acetabular reconstruction in total hip arthroplasty for developmental dysplasia of the hip with complicated deformity]. Peng Y, Yang L, Chen G, Gu L, Chen H. Zhonghua Wai Ke Za Zhi. 2014 Jan;52(1):25-9. Chinese. PMID: 24697936.
3. [Retrospective analysis on total hip arthroplasty for the treatment of developmental dysplasia of the hip in 29 adults]. Cao YS, Lu M, Yao GH, Li WN, Zhu FP, Zhang B. Zhongguo Gu Shang. 2013 Nov;26(11):962-5. Chinese. PMID: 24605754
4. A Reduction Technique of Arthroplasty Without Subtrochanteric Femoral Shortening Osteotomy for the Treatment of Developmental High Dislocation of Hip: A Case Series of 28 Hips. Yan F, Chen G, Yang L, He R, Gu L, Wang F. J Arthroplasty. 2013 Dec 4. pii: S0883-5403(13)00861-9. doi: 10.1016/j.arth.2013.11.016. [Epub ahead of print] PMID: 24412147
5. [Total hip arthroplasty for treatment of Crowe type IV congenital dysplasia of hip with dislocation in adults]. Li W, Zhang W, Bai G, Huang Z, Shen R. Zhongguo Xiu Fu Chong Jian Wai Ke Za Zhi. 2013 Oct;27(10):1153-6. Chinese. PMID: 24397121
6. [Becker V-shaped lateral rotation osteotomy in total hip arthroplasty for Crowe type IV development dislocation of hip]. Li Y, Ma W, Sun J, Song X, An M, Zhang Q. Zhongguo Xiu Fu Chong Jian Wai Ke Za Zhi. 2013 Sep;27(9):1032-6. Chinese. PMID: 24279008
7. Subtrochanteric shortening osteotomy combined with cemented total hip arthroplasty for Crowe group IV hips. Oe K, Iida H, Nakamura T, Okamoto N, Wada T. Arch Orthop Trauma Surg. 2013 Dec;133(12):1763-70. doi: 10.1007/s00402-013-1869-4. PMID: 24121623
8. Subtrochanteric shortening in total hip arthroplasty: biomechanical comparison of four techniques. Muratli KS, Karatosun V, Uzun B, Celik S. J Arthroplasty. 2014 Apr;29(4):836-42. doi: 10.1016/j.arth.2013.09.004. Epub 2013 Oct 4. PMID: 24095585
9. Total hip arthroplasty with subtrochanteric shortening osteotomy for Crowe grade 4 dysplasia using the direct anterior approach. Oinuma K, Tamaki T, Miura Y, Kaneyama R, Shiratsuchi H. J Arthroplasty. 2014 Mar;29(3):626-9. doi: 10.1016/j.arth.2013.07.038. Epub 2013 Aug 30. PMID: 23998992
10. Total hip arthroplasty after treatment of pseudojoint infection in a patient with a highly dislocated hip. Park KS, Seon JK, Nah SY, Yoon TR. Case Rep Orthop. 2013;2013:947121. doi: 10.1155/2013/947121. Epub 2013 Jun 25. PMID: 23878754
11. [Total hip arthroplasty for crowe type IV developmental dysplasia of the hip with S-ROM prosthesis]. Sun QC, Wang XH, Song BS, Zhu FB, Yan SG. Zhongguo Gu Shang. 2013 Feb;26(2):153-7. Chinese. PMID: 23678766
12. Midterm results after subtrochanteric end-to-side valgization osteotomy in severe infantile coxa vara. Günther CM, Komm M, Jansson V, Heimkes B. J Pediatr Orthop. 2013 Jun;33(4):353-60. doi: 10.1097/BPO.0b013e3182812194. PMID: 23653021
13. Waking up the healing potential of the bone:subtrochanteric non-union in femoral dysplasia successfully treated after 40 years of pain and disability. Kloen P, Nützinger J. BMJ Case Rep. 2013 Jan 3;2013. pii: bcr2012007469. doi: 10.1136/bcr-2012-007469. PMID: 23291811
14. Predicting leg-length change after total hip arthroplasty by measuring preoperative hip flexion under general anaesthesia. Fujishiro T, Nishiyama T, Hayashi S, Hashimoto S, Kurosaka M, Kanno T, Masuda T. J Orthop Surg (Hong Kong). 2012 Dec;20(3):327-30. PMID: 23255639
15. Cementless total hip replacement for severe developmental dysplasia of the hip: our experience in Crowe's group IV. Imarisio D, Trecci A, Sabatini L, Uslenghi M, Leone C, Scagnelli R. Musculoskelet Surg. 2013 Apr;97(1):25-30. doi: 10.1007/s12306-012-0227-y. Epub 2012 Oct 14. PMID: 23065630
16. Total hip arthroplasty with shortening subtrochanteric Z osteotomy in the treatment of developmental dysplasia with high hip dislocation. Semenowicz J, Szymański S, Walo R, Czuma P, Pijet B. Ortop Traumatol Rehabil. 2012 Jul-Aug;14(4):341-9. doi: 10.5604/15093492.1005094. PMID: 23043057
17. Cementless total hip arthroplasty with modified oblique femoral shortening osteotomy in Crowe type IV congenital hip dislocation. Kiliçoğlu Oİ, Türker M, Akgül T, Yazicioğlu O. J Arthroplasty. 2013 Jan;28(1):117-25. doi: 10.1016/j.arth.2012.06.014. Epub 2012 Aug 3. PMID: 22868069
18. Treatment of high hip dislocation with a cementless stem combined with a shortening osteotomy. Baz AB, Senol V, Akalin S, Kose O, Guler F, Turan A. Arch Orthop Trauma Surg. 2012 Oct;132(10):1481-6. Epub 2012 Jun 10. PMID: 22684740
19. [Analysis of perioperation complications of total hip arthroplasty in treating Crowe type IV developmental dysplasia of the hip]. Li JY, Guan GH, Li XF, Huang S, Wu M, Gao HL, Sun JY. Zhongguo Gu Shang. 2012 Jan;25(1):74-7. Chinese. PMID: 22489531
20. Leg length change in total hip arthroplasty with subtrochanteric femoral shortening osteotomy for Crowe type IV developmental hip dysplasia. Fujishiro T, Nishiyama T, Hayashi S, Kurosaka M, Kanno T, Masuda T. J Arthroplasty. 2012 Jun;27(6):1019-22. doi: 10.1016/j.arth.2012.01.032. Epub 2012 Apr 3. PMID: 22480527
21. S-ROM modular arthroplasty combined with transverse subtrochanteric shortening for Crowe type IV congenital dislocation of hip. Zhong C, Cai XZ, Yan SG, He RX. Chin Med J (Engl). 2011 Dec;124(23):3891-5. PMID: 22340315
22. [Femoral reconstruction in patients with proximal femoral deformity in total hip arthroplasty]. Li F, Tian H, Zhang K, Liu Y. Zhongguo Xiu Fu Chong Jian Wai Ke Za Zhi. 2011 Oct;25(10):1188-91. Chinese. PMID: 22069971
23. Dislocation after total hip arthroplasty among patients with developmental dysplasia of the hip. Wang L, Trousdale RT, Ai S, An KN, Dai K, Morrey BF. J Arthroplasty. 2012 May;27(5):764-9. doi: 10.1016/j.arth.2011.08.021. Epub 2011 Oct 21. PMID: 22018536
24. [Total hip arthroplasty with shortening subtrochanteric osteotomy and custom-made prosthesis in Crowe type IV developmental dysplasia]. Starker M, Bischof F, Lindenfeld T. Z Orthop Unfall. 2011 Oct;149(5):518-25. doi: 10.1055/s-0031-1280029. Epub 2011 Oct 7. German. PMID: 21984422
25. Total hip arthroplasty with shortening osteotomy in congenital major hip dislocation sequelae. Dallari D, Pignatti G, Stagni C, Giavaresi G, Del Piccolo N, Rani N, Veronesi F, Fini M. Orthopedics. 2011 Aug 8;34(8):e328-33. doi: 10.3928/01477447-20110627-14. PMID: 21815571
26. Cemented total hip arthroplasty with subtrochanteric femoral shortening transverse osteotomy for severely dislocated hips: outcome with a 3- to 10-year follow-up period. Akiyama H, Kawanabe K, Yamamoto K, Kuroda Y, So K, Goto K, Nakamura T. J Orthop Sci. 2011 May;16(3):270-7. doi: 10.1007/s00776-011-0049-z. Epub 2011 Mar 26. PMID: 21442186
27. Cementless modular total hip arthroplasty with subtrochanteric shortening osteotomy for hips with developmental dysplasia. Takao M, Ohzono K, Nishii T, Miki H, Nakamura N, Sugano N. J Bone Joint Surg Am. 2011 Mar 16;93(6):548-55. doi: 10.2106/JBJS.I.01619. PMID: 21411705
28. Treatment of Crowe IV high hip dysplasia with total hip replacement using the Exeter stem and shortening derotational subtrochanteric osteotomy. Charity JA, Tsiridis E, Sheeraz A, Howell JR, Hubble MJ, Timperley AJ, Gie GA. J Bone Joint Surg Br. 2011 Jan;93(1):34-8. doi: 10.1302/0301-620X.93B1.24689. PMID: 21196540
29. Gait analysis in adults with severe hip dysplasia before and after total hip arthroplasty. Marangoz S, Atilla B, Gök H, Yavuzer G, Ergin S, Tokgözoğlu AM, Alpaslan M. Hip Int. 2010 Oct-Dec;20(4):466-72. PMID: 21157751
30. Total hip arthroplasty with shortening subtrochanteric osteotomy in Crowe type-IV developmental dysplasia: surgical technique. Krych AJ, Howard JL, Trousdale RT, Cabanela ME, Berry DJ. J Bone Joint Surg Am. 2010 Sep;92 Suppl 1 Pt 2:176-87. doi: 10.2106/JBJS.J.00061. PMID: 20844173
31. Cemented total hip arthroplasty with subtrochanteric osteotomy in dysplastic hips. Howie CR, Ohly NE, Miller B. Clin Orthop Relat Res. 2010 Dec;468(12):3240-7. doi: 10.1007/s11999-010-1367-8 PMID: 20461484
32. Cementless total hip arthroplasty with subtrochanteric transverse shortening osteotomy for severely dysplastic or dislocated hips. Yalcin N, Kilicarslan K, Karatas F, Mutlu T, Yildirim H. Hip Int. 2010 Jan-Mar;20(1):87-93. PMID: 20235079
33. Femoral shortening in total hip arthroplasty for high developmental dysplasia of the hip. Reikerås O, Haaland JE, Lereim P. Clin Orthop Relat Res. 2010 Jul;468(7):1949-55. doi: 10.1007/s11999-009-1218-7. Epub 2010 Jan 14. PMID: 20077043
34. Large diameter metal-on-metal total hip arthroplasty for Crowe IV developmental dysplasia of the hip. Parmaksizoglu AS, Ozkaya U, Bilgili F, Basilgan S, Kabukcuoglu Y. Hip Int. 2009 Oct-Dec;19(4):309-14. PMID: 20041376
35. Retracted: Double chevron subtrochanteric shortening derotational osteotomy in cementless total hip arthroplasty for Crowe type IV congenital dislocation of the hip. [No authors listed] J Arthroplasty. 2011 Feb;26(2):340. doi: 10.1016/j.arth.2009.08.006. Epub 2009 Oct 17. No abstract available. PMID: 19837553
36. Total hip arthroplasty with shortening subtrochanteric osteotomy in Crowe type-IV developmental dysplasia. Krych AJ, Howard JL, Trousdale RT, Cabanela ME, Berry DJ. J Bone Joint Surg Am. 2009 Sep;91(9):2213-21. doi: 10.2106/JBJS.H.01024. PMID: 19723999
37. Cementless total hip replacement with subtrochanteric femoral shortening for severe developmental dysplasia of the hip. Nagoya S, Kaya M, Sasaki M, Tateda K, Kosukegawa I, Yamashita T. J Bone Joint Surg Br. 2009 Sep;91(9):1142-7. doi: 10.1302/0301-620X.91B9. 21736. PMID: 19721037
38. A new technique of subtrochanteric shortening in total hip replacement for Crowe type 3 to 4 dysplasia of the hip. Togrul E, Ozkan C, Kalaci A, Gülşen M. J Arthroplasty. 2010 Apr;25(3):465-70. doi: 10.1016/j.arth.2009.02.023. Epub 2009 Jul 4. PMID: 19577893
39. [Subtrochanteric end-to-side valgus osteotomy for severe infantile coxa vara]. Heimkes B, Komm M, Melcher C. Oper Orthop Traumatol. 2009 Mar;21(1):97-111. doi: 10.1007/s00064-009-1609-7. German.PMID: 19326071
40. [Pemberton acetabuloplasty for treating the developmental dislocation of hip joint]. Wu TN, Guo HY, Zhang YF. Zhongguo Gu Shang. 2008 Sep;21(9):681-3. Chinese. PMID: 19105282
41. [Soft tissue balancing in the total hip arthroplasty for severe developmental dysplasia of the hip in adults]. Zhang L, Yu LD, Yang GJ. Zhonghua Wai Ke Za Zhi. 2008 Sep 1;46(17):1299-302. Chinese. PMID: 19094558
42. [A comparative study of the role of two femoral shortening techniques in total hip arthroplasty on patients with Crowe's IV congenital dislocated hips]. Zhou YG, Zhang Q, Wang Y, Chen C, Chen JY, Zhang GQ, Hao LB, Li JD, Dong JY, Lin F. Zhonghua Wai Ke Za Zhi. 2008 Sep 1;46(17):1288-92. Chinese. PMID: 19094555
43. Distal femoral shortening in total hip arthroplasty for complex primary hip reconstruction. A new surgical technique. Koulouvaris P, Stafylas K, Sculco T, Xenakis T. J Arthroplasty. 2008 Oct;23(7):992-8. doi: 10.1016/j.arth.2007.09.013. Epub 2008 Mar 4. PMID: 18534497
44. [Is there a need of an additional extramedullary fixation in transverse subtrochanteric shortening in primary total hip arthroplasty for patients with severe hip dysplasia? Short-term experience in seven patients with congenital dislocation]. Götze C, Winkelmann W, Gosheger G, Rödl R. Z Orthop Unfall. 2007 Sep-Oct;145(5):568-73. German. PMID: 17939065
45. Subtrochanteric valgus osteotomy for chronically dislocated, painful spastic hips. Surgical technique. Hogan KA, Blake M, Gross RH. J Bone Joint Surg Am. 2007 Sep;89 Suppl 2 Pt.2:226-31. PMID: 17768217
46. Total hip arthroplasty requiring subtrochanteric osteotomy for developmental hip dysplasia: 5- to 14-year results. Bernasek TL, Haidukewych GJ, Gustke KA, Hill O, Levering M. J Arthroplasty. 2007 Sep;22(6 Suppl 2):145-50. Epub 2007 Jul 27. PMID: 17823034
47. Transverse subtrochanteric shortening osteotomy in primary total hip arthroplasty for patients with severe hip developmental dysplasia. Park MS, Kim KH, Jeong WC. J Arthroplasty. 2007 Oct;22(7):1031-6. PMID: 17920477
48. Cemented femoral stems in patients with DDH. Garcia-Cimbrelo E. Hip Int. 2007;17 Suppl 5:S128-33. PMID: 19197894
49. Evolution of surgical techniques for the treatment of angular and torsional deviation in DDH: 20 years experience. Grappiolo G, Spotorno L, Burastero G. Hip Int. 2007;17 Suppl 5:S105-10. PMID: 19197890
50. Subtrochanteric valgus osteotomy for chronically dislocated, painful spastic hips. Hogan KA, Blake M, Gross RH. J Bone Joint Surg Am. 2006 Dec;88(12):2624-31. PMID: 17142412
51. The S-rOM modular femoral stem in dysplasia of the hip. Mattingly DA. Orthopedics. 2005 Sep;28(9 Suppl):s1069-73. Review. PMID: 16190039
52. Surgical treatment of developmental dysplasia of the hip in the periadolescent period. Papavasiliou VA, Papavasiliou AV. J Orthop Sci. 2005;10(1):15-21. PMID: 15666117
53. Subtrochanteric shortening and derotational osteotomy in primary total hip arthroplasty for patients with severe hip dysplasia: 5-year follow-up. Masonis JL, Patel JV, Miu A, Bourne RB, McCalden R, Macdonald SJ, Rorabeck CH. J Arthroplasty. 2003 Apr;18(3 Suppl 1):68-73. PMID: 12730932
54. [Cemented total hip arthroplasty for severe dysplasia or congenital dislocation of the hip]. Oztürkmen Y, Karli M, Doğrul C. Acta Orthop Traumatol Turc. 2002;36(3):195-202. Turkish. PMID: 12510076
55. Surgical treatment of developmental dysplasia of the hip in adults: II. Arthroplasty options. Sanchez-Sotelo J, Berry DJ, Trousdale RT, Cabanela ME. J Am Acad Orthop Surg. 2002 Sep-Oct;10(5):334-44. Review. PMID: 12374484
56. [Total hip arthroplasty after proximal femoral osteotomy: 75 cases with 9-year follow-up]. Delbarre JC, Hulet C, Schiltz D, Aubriot JH, Vielpeau C. Rev Chir Orthop Reparatrice Appar Mot. 2002 May;88(3):245-56. French. PMID: 12037480
57. Perthes' disease or late avascular necrosis after developmental dislocation of the hip? 10 children followed for 6-35 years. Koczewski P, Napiontek M. Acta Orthop Scand. 2001 Aug;72(4):331-4. PMID: 11580119
58. A new technique of subtrochanteric shortening in total hip arthroplasty: surgical technique and results of 9 cases. Bruce WJ, Rizkallah SM, Kwon YM, Goldberg JA, Walsh WR. J Arthroplasty. 2000 Aug;15(5):617-26. PMID: 10960001
59. Subtrochanteric valgus-extension osteotomy for neglected congenital dislocation of the hip in young adults. Aksoy MC, Musdal Y. Acta Orthop Belg. 2000 Apr;66(2):181-6. PMID: 10842880
60. Uncemented total hip arthroplasty with subtrochanteric derotational osteotomy for severe femoral anteversion. Zadeh HG, Hua J, Walker PS, Muirhead-Allwood SK. J Arthroplasty. 1999 Sep;14(6):682-8. PMID: 10512440
61. Treatment of congenital dislocated hip by arthroplasty with femoral shortening. Chareancholvanich K, Becker DA, Gustilo RB. Clin Orthop Relat Res. 1999 Mar;(360):127-35. PMID: 10101318
62. Avulsion fracture of the ischium following complex total hip arthroplasty: an unusual cause of hip pain. Smith PN, Gie GA. J Arthroplasty. 1998 Aug;13(5):603-6. PMID: 9726330
63. Subtrochanteric femoral shortening osteotomy in total hip arthroplasty for high-riding developmental dislocation of the hip. Yasgur DJ, Stuchin SA, Adler EM, DiCesare PE. J Arthroplasty. 1997 Dec;12(8):880-8. PMID: 9458253
64. Intra-articular hip arthrodesis without subtrochanteric osteotomy in adolescents: technique and short-term follow-up. Schoenecker PL, Johnson LO, Martin RA, Doyle P, Capelli AM. Am J Orthop (Belle Mead NJ). 1997 Apr;26(4):257-64. PMID: 9113292
65. Femoral remodelling after subtrochanteric osteotomy for developmental dysplasia of the hip. Sangavi SM, Szöke G, Murray DW, Benson MK. J Bone Joint Surg Br. 1996 Nov;78(6):917-23. PMID: 8951007
66. [Long-term outcome of Dega acetabulum-plasty]. Reichel H, Haunschild M, Hein W. Z Orthop Ihre Grenzgeb. 1996 Mar-Apr;134(2):131-6. German. PMID: 8779256
67. Late results after subtrochanteric angulation osteotomy in young patients. Schiltenwolf M, Carstens C, Bernd L, Lukoschek M. J Pediatr Orthop B. 1996 Fall;5(4):259-67. PMID: 8897259
68. Double-chevron subtrochanteric shortening derotational femoral osteotomy combined with total hip arthroplasty for the treatment of complete congenital dislocation of the hip in the adult. Preliminary report and description of a new surgical technique. Becker DA, Gustilo RB. J Arthroplasty. 1995 Jun;10(3):313-8. PMID: 7673910
69. [Results of surgical repositioning of congenital hip dislocation]. Dungl P, Grill F, Cechová I. Acta Chir Orthop Traumatol Cech. 1993;60(6):324-33. Czech. PMID: 8128808
70. Pelvic support femoral reconstruction using the method of Ilizarov: a case report. Samchukov ML, Birch JG. Bull Hosp Jt Dis. 1992 Summer;52(1):7-11. PMID: 1422442
71. Varus rotational femoral osteotomies in adults with hip dysplasia. Pellicci PM, Hu S, Garvin KL, Salvati EA, Wilson PD Jr. Clin Orthop Relat Res. 1991 Nov;(272):162-6. PMID: 1934728
72. [Total hip prosthesis after high femoral osteotomy]. Carret JP, Dejour H, Biancarelli P, Bonnin M, Galland O. Rev Chir Orthop Reparatrice Appar Mot. 1991;77(2):83-91. Review. French. PMID: 1829252
73. Osteotomy for femoral anteversion. A prospective 9-year study of 52 children. Svenningsen S, Terjesen T, Apalset K, Anda S. Acta Orthop Scand. 1990 Aug;61(4):360-3. PMID: 2402992
74. [Surgical treatment of residual congenital dysplasia with dislocation of the hip joint after treatment with the Frejka pillow]. Lempicki A, Wierusz-Kozłowska M. Chir Narzadow Ruchu Ortop Pol. 1990;55(1):37-42. Polish. PMID: 1369921
75. Subtrochanteric osteotomy with intramedullary fixation for arthroplasty of the dysplastic hip. A case report. Sponseller PD, McBeath AA. J Arthroplasty. 1988;3(4):351-4. PMID: 3241173
76. [Experience with subtrochanteric osteotomy by hyperextension-abduction in the management of bilateral congenital hip dislocation]. Gurin J. Magy Traumatol Orthop Helyreallito Seb. 1987;30(2):97-102. Hungarian. No abstract available. PMID: 2884364
77. Idiopathic increased anteversion of the femoral neck. Radiological and clinical study in non-operated and operated patients. Reikerås O, Bjerkreim I. Acta Orthop Scand. 1982 Dec;53(6):839-45. PMID: 7180393
78. Closure of the epiphysis of the femoral head and of the triradiate cartilage of the acetabulum following surgery for congenital hip dislocation. Makin M. Isr J Med Sci. 1980 Apr;16(4):307-10. PMID: 7390781
79. Reduction of neglected congenital dislocation of the hip in children over the age of six years. Herold HZ, Daniel D. J Bone Joint Surg Br. 1979 Feb;61(1):1-6. PMID: 422627
80. [Subtrochanteric osteotomy of the hip with creation of a double "catch" in treatment of congenital dislocation of the hip]. Sadyrbaev KS, Makazhanov KhZh. Ortop Travmatol Protez. 1976 Sep;(9):68-70. Russian. No abstract available. PMID: 1012697
81. The treatment of developmental coxa vara by abduction subtrochanteric and intertrochanteric femoral osteotomy with special reference to the role of adductor tenotomy. Weighill FJ. Clin Orthop Relat Res. 1976 May;(116):116-24. PMID: 1277629
82. Subtrochanteric derotational femoral osteotomy (author's transl)]. Fait M, Janovec M. Acta Chir Orthop Traumatol Cech. 1976 Feb;43(1):58-61. Czech. No abstract available. PMID: 1266530
83. Acetabuloplasty in the treatment of congenital dislocation of the hip. Trevor D, Johns DL, Fixsen JA. J Bone Joint Surg Br. 1975 May;57(2):167-74. PMID: 1095588
84. [Subtrochanteric osteotomy in the treatment of inveterate congenital hip dislocation (long term control of 60 interventions)]. Stringa G, Aglietti P. Arch Putti Chir Organi Mov. 1971;26:165-83. Italian. No abstract available. PMID: 5155652
85. [Varus producing and derotational subtrochanteric osteotomy in the treatment of congenital dislocation of the hip]. Shimazu A, Fukunishi O. Shujutsu. 1970 Jun;24(6):695-701. Japanese. No abstract available. PMID: 5447611
86. [Bone screw for bone fragment fixation after subtrochanteric osteotomy in the treatment of congenital hip dislocation]. Szczekot J. Chir Narzadow Ruchu Ortop Pol. 1970;35(4):485-8. Polish. No abstract available. PMID: 5528386
87. [Subtrochanteric fatigue fracture in old high hip dislocation]. Mau H. Z Orthop Ihre Grenzgeb. 1967 Oct;103(4):537-9. German. No abstract available. PMID: 4232934
88. [Modification of subtrochanteric oblique osteotomy of the femur in reconstruction-reposition of congenital dislocation of the hip]. Marciniak W. Chir Narzadow Ruchu Ortop Pol. 1967;32(4):527-9. Polish. No abstract available. PMID: 6058877
89. VARUS-PRODUCING AND DEROTATIONAL SUBTROCHANTERIC OSTEOTOMY IN THE TREATMENT OF CONGENITAL DISLOCATION OF THE HIP. CHUINARD EG, LOGAN ND. J Bone Joint Surg Am. 1963 Oct;45:1397-408. No abstract available. PMID: 14069779
90. [Measured subtrochanteric detorsive osteotomy in reducing congenital hip dislocation]. TELESZYNSKI M. Chir Narzadow Ruchu Ortop Pol. 1962;27:153-62. Polish. No abstract available. PMID: 13920137
91. [On the treatment of inveterate congenital bilateral hip luxations with subtrochanteric hyperextension osteotomy]. GLAUBER A. Z Orthop Ihre Grenzgeb. 1960;93:502-8. German. No abstract available. PMID: 13705979
92. [Subtrochanteric osteotomy in inveterate congenital dislocation of the hip]. BARBIERI E, FIUME M. Arch Ortop. 1959;72(1):128-40. Italian. No abstract available. PMID: 13650927
93. [Follow-up of Mommsen subtrochanteric osteotomy in coxa vara secondary to bloodlessly reduced congenital luxation of the hip]. COZZOLINO A. Minerva Ortop. 1954 Dec;5(12):524-5. Italian. No abstract available. PMID: 14355978
94. Cementless total hip replacement with subtrochanteric femoral shortening for severe developmental dysplasia of the hip. Decking J, Decking R, Schoellner C, Fuerderer S, Rompe JD, Eckardt A. Arch Orthop Trauma Surg. 2003 Sep;123(7):357-62. Epub 2003 Jul 3. PMID: 12844229
95. Total hip arthroplasty in developmental high dislocation of the hip. Erdemli B, Yilmaz C, Atalar H, Güzel B, Cetin I. J Arthroplasty. 2005 Dec;20(8):1021-8. PMID: 16376258
96. Total hip arthroplasty for Crowe type IV developmental dysplasia. Hasegawa Y, Iwase T, Kanoh T, Seki T, Matsuoka A. J Arthroplasty. 2012 Oct;27(9):1629-35. doi: 10.1016/j.arth.2012.02.026. Epub 2012 Apr 30. PMID: 22552220
97. Cementless total hip arthroplasty for dysplastic and dislocated hips. Kılıçarslan K, Yalçın N, Karataş F, Catma F, Yıldırım H. Eklem Hastalik Cerrahisi. 2011;22(1):8-15. PMID: 21417980
98. Total hip arthroplasty in adult patients who had developmental dysplasia of the hip. Kim YH, Kim JS. J Arthroplasty. 2005 Dec;20(8):1029-36. PMID: 16376259
99. Results on total hip arthroplasties with femoral shortening for Crowe's group IV dislocated hips. Makita H, Inaba Y, Hirakawa K, Saito T. J Arthroplasty. 2007 Jan;22(1):32-8.PMID: 17197306
100. Femoral shortening in total arthroplasty for completely dislocated hips: 3-7 year results in 25 cases. Reikeraas O, Lereim P, Gabor I, Gunderson R, Bjerkreim I. Acta Orthop Scand. 1996 Feb;67(1):33-6. PMID: 8615099
101. Femoral shortening and cementless arthroplasty in high congenital dislocation of the hip. Sener N, Tözün IR, Aşik M. J Arthroplasty. 2002 Jan;17(1):41-8. PMID: 11805923
102. Cemented total hip arthroplasty with transverse subtrochanteric shortening osteotomy for Crowe group IV dislocated hip. Kawai T, Tanaka C, Ikenaga M, Kanoe H. J Arthroplasty. 2011 Feb;26(2):229-35. doi: 10.1016/j.arth.2010.03.029. Epub 2010 May 31. PMID: 20570099
