# Supplementary figures and images for: Comparison of transverse and modified subtrochanteric femoral shortening osteotomy in total hip arthroplasty for developmental dysplasia of hip: a meta-analysis
Source: BMC Musculoskelet Disord. 2014 Oct 3;15:331. doi: 10.1186/1471-2474-15-331 (PMC4201680; doi:10.1186/1471-2474-15-331)

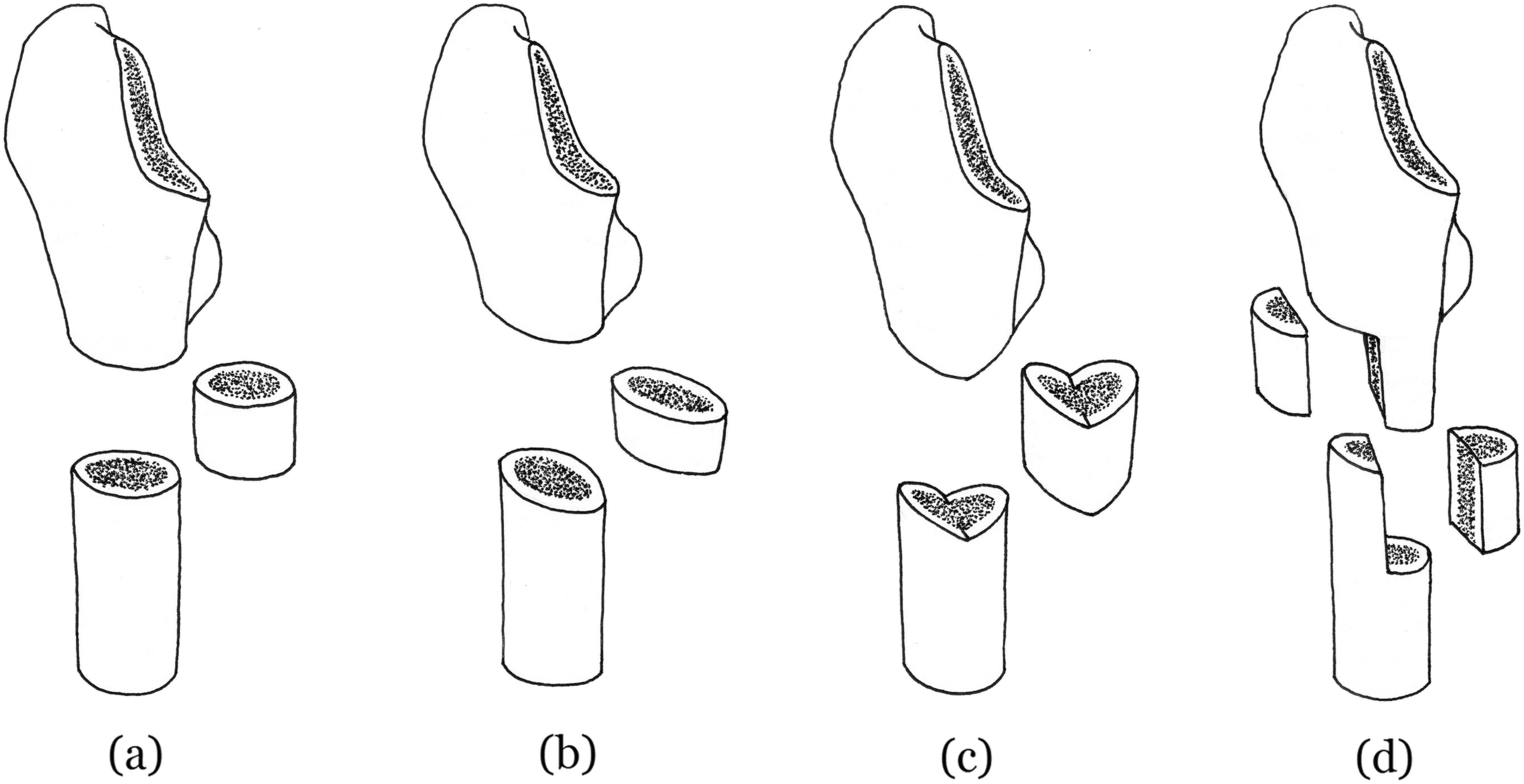

Supplement: Supplementary file 2 — Authors’ original file for figure 1 [file 12891_2014_2280_MOESM2_ESM.tif]

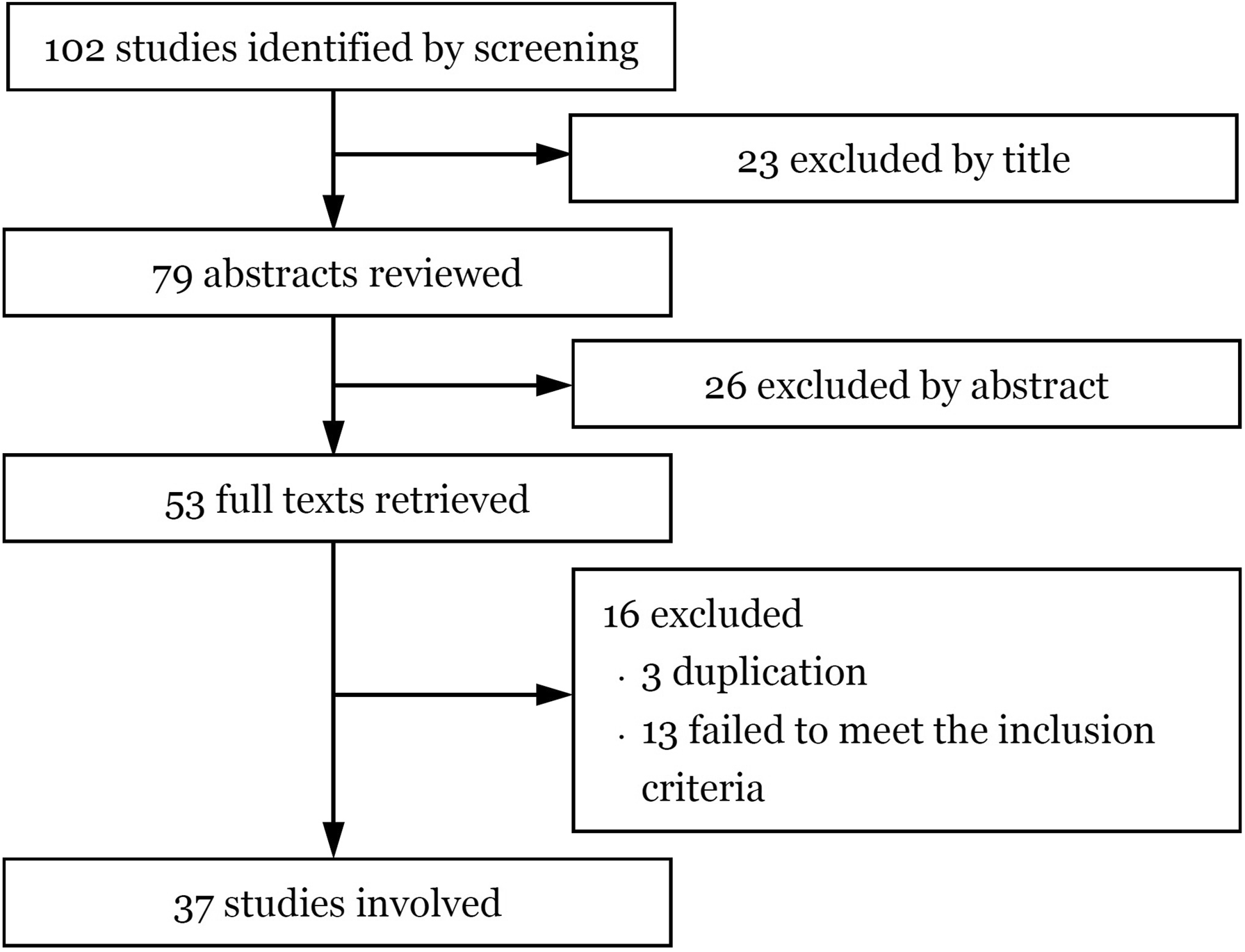

Supplement: Supplementary file 3 — Authors’ original file for figure 2 [file 12891_2014_2280_MOESM3_ESM.tif]

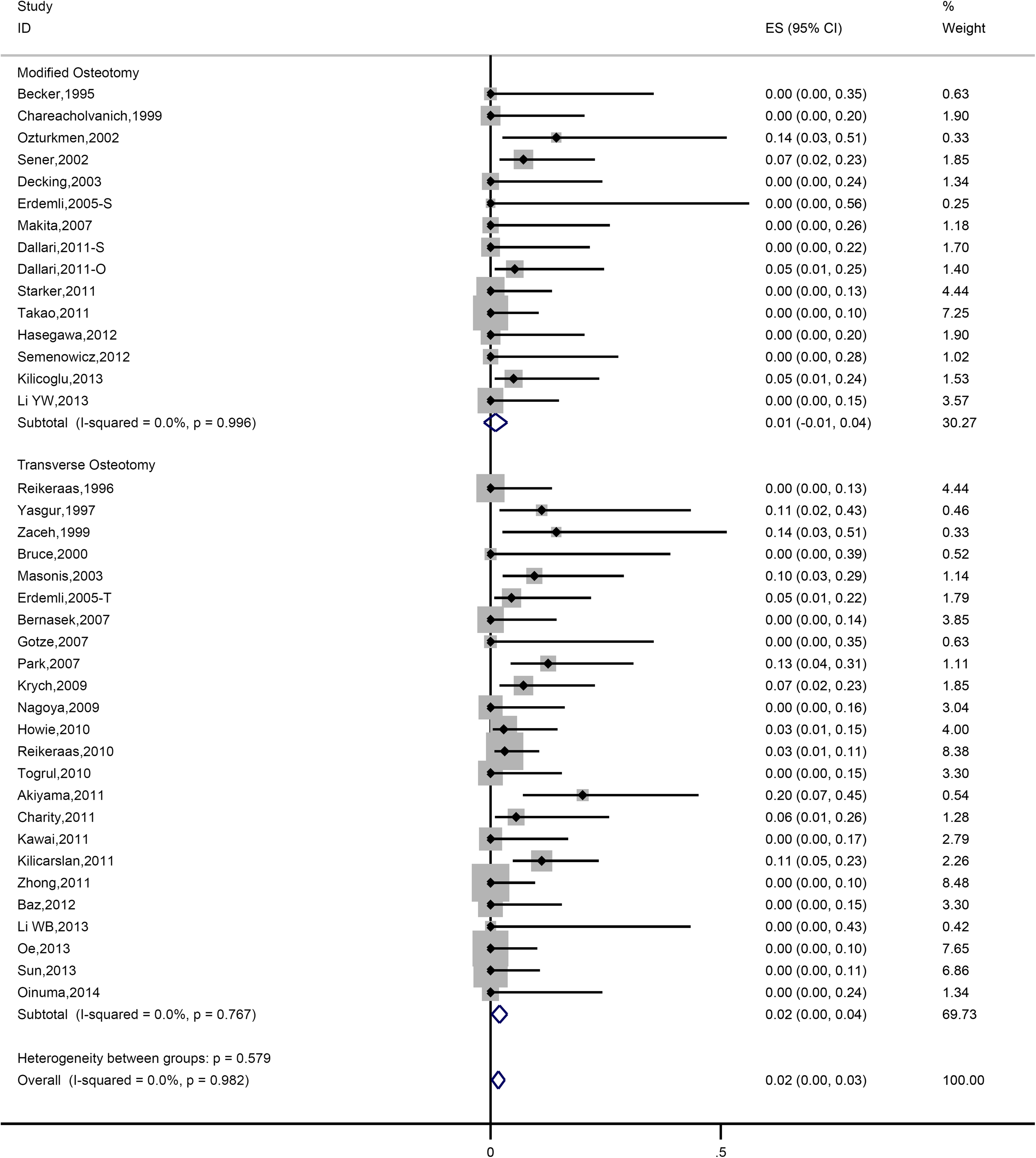

Supplement: Supplementary file 4 — Authors’ original file for figure 3 [file 12891_2014_2280_MOESM4_ESM.tif]

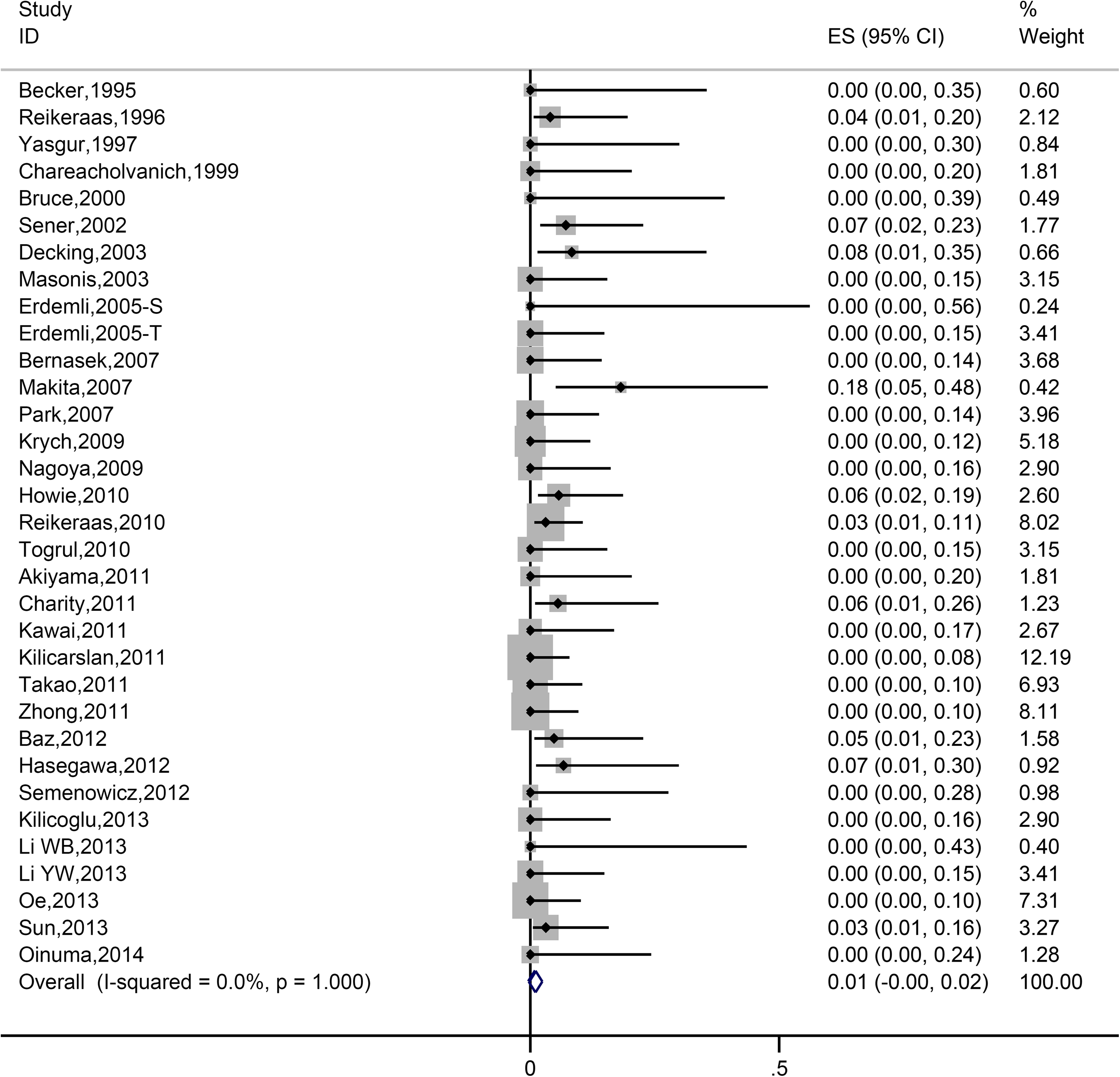

Supplement: Supplementary file 5 — Authors’ original file for figure 4 [file 12891_2014_2280_MOESM5_ESM.tif]

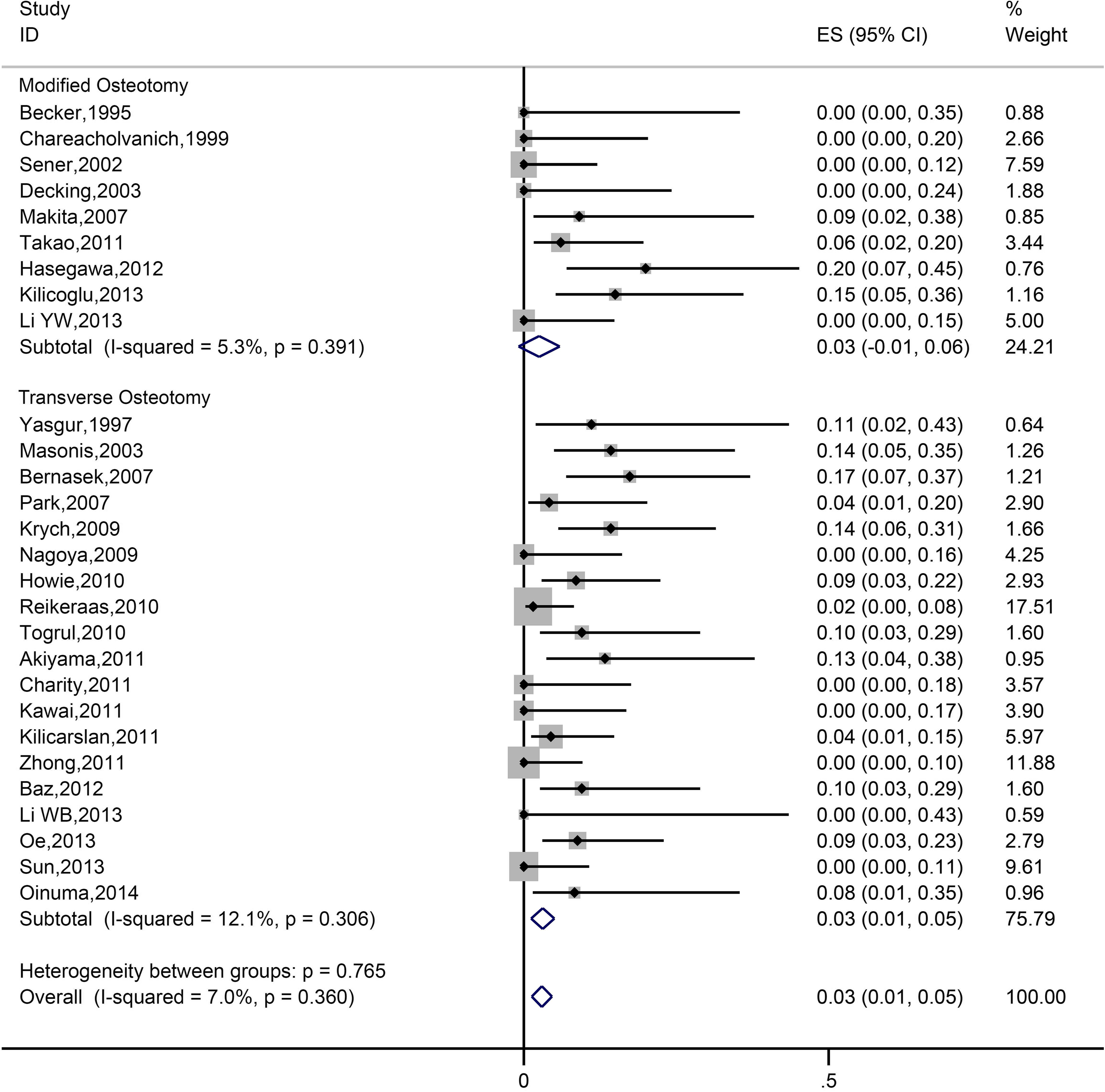

Supplement: Supplementary file 6 — Authors’ original file for figure 5 [file 12891_2014_2280_MOESM6_ESM.tif]

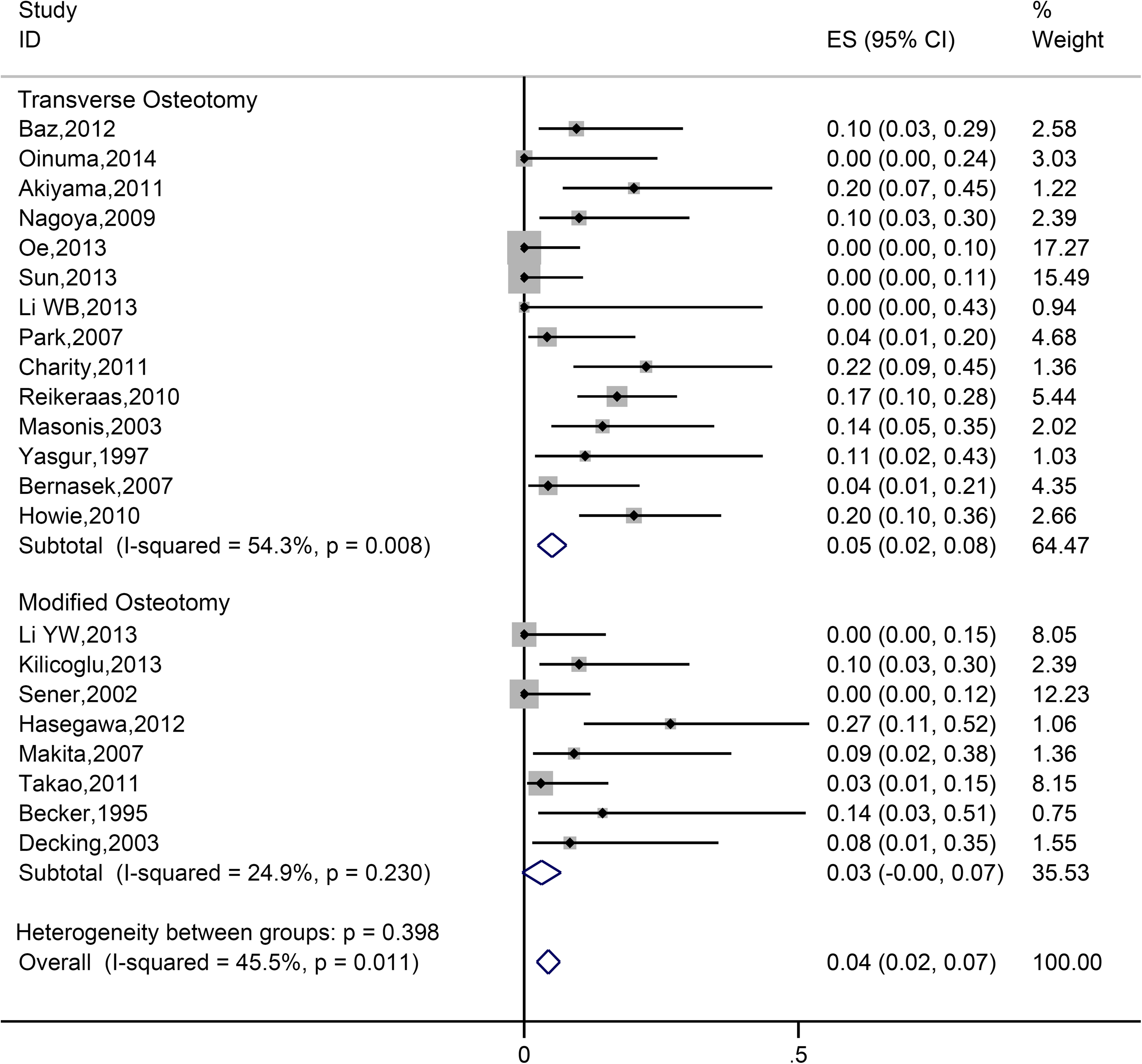

Supplement: Supplementary file 7 — Authors’ original file for figure 6 [file 12891_2014_2280_MOESM7_ESM.tif]

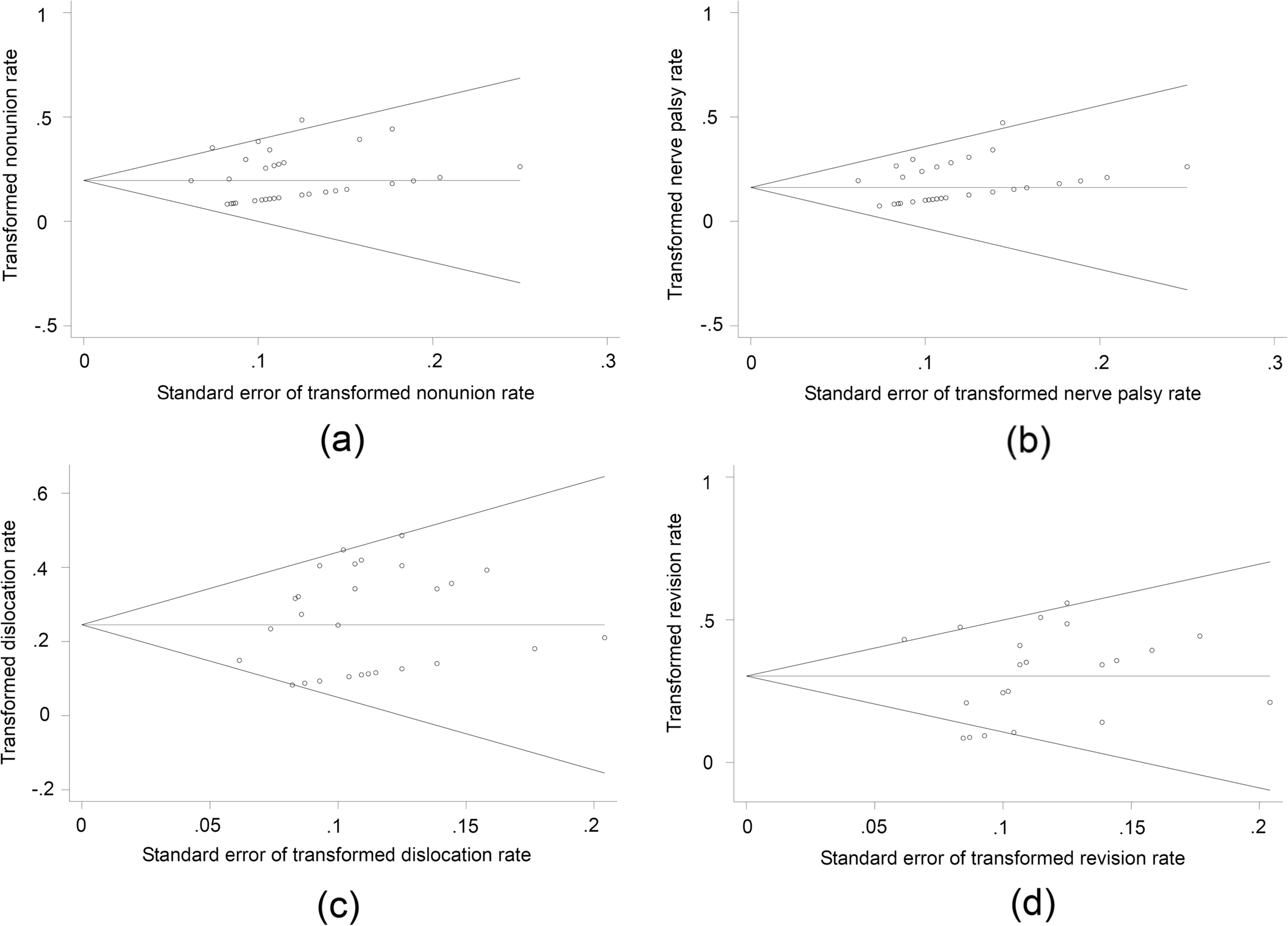

Supplement: Supplementary file 8 — Authors’ original file for figure 7 [file 12891_2014_2280_MOESM8_ESM.tif]

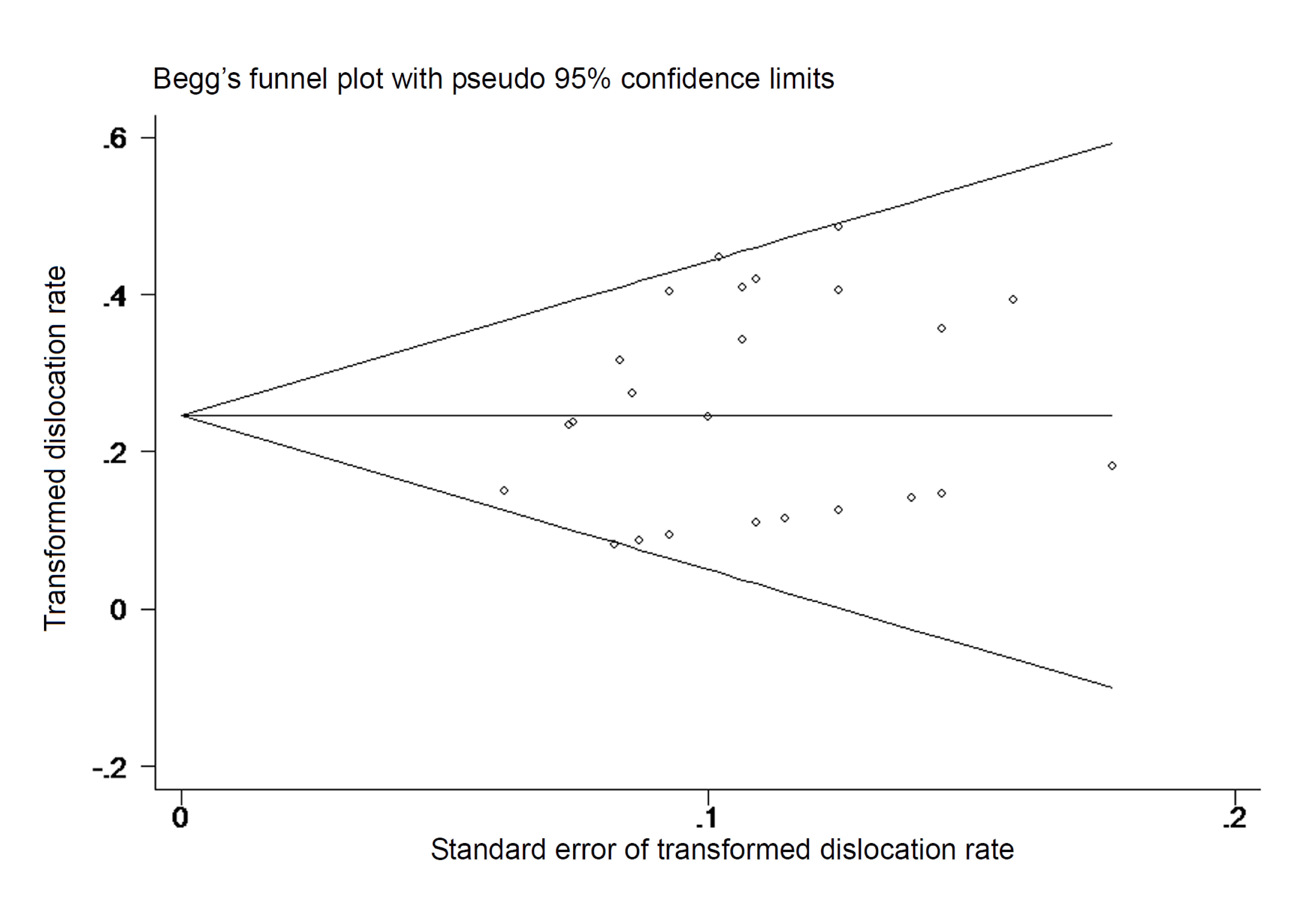

Supplement: Supplementary file 9 — Authors’ original file for figure 8 [file 12891_2014_2280_MOESM9_ESM.tiff]

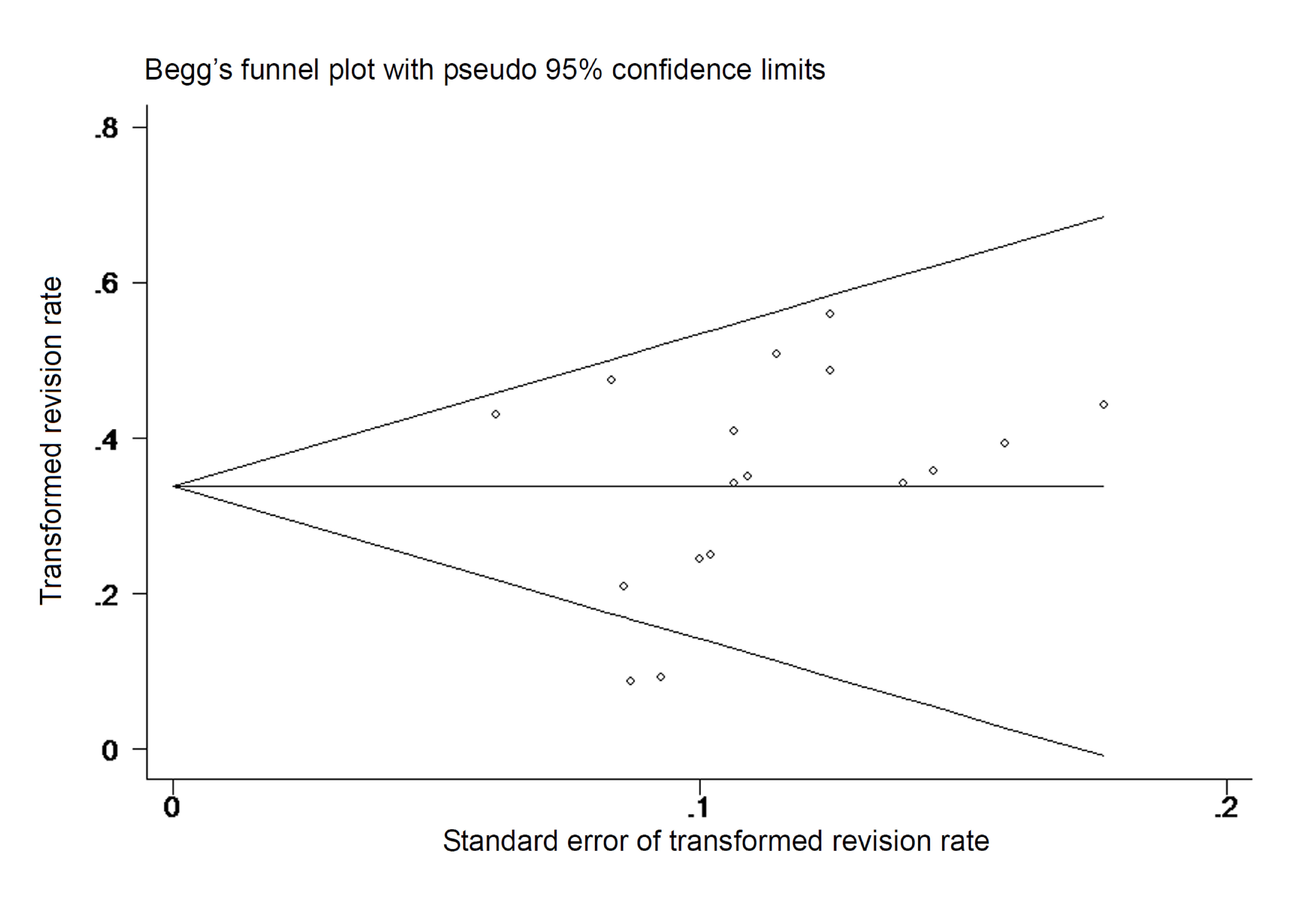

Supplement: Supplementary file 10 — Authors’ original file for figure 9 [file 12891_2014_2280_MOESM10_ESM.tiff]
